# Targeting cancer cell plasticity by HDAC inhibition to reverse EBV-induced dedifferentiation in nasopharyngeal carcinoma

**DOI:** 10.1038/s41392-021-00702-4

**Published:** 2021-09-04

**Authors:** Jiajun Xie, Zifeng Wang, Wenjun Fan, Youping Liu, Fang Liu, Xiangbo Wan, Meiling Liu, Xuan Wang, Deshun Zeng, Yan Wang, Bin He, Min Yan, Zijian Zhang, Mengjuan Zhang, Zhijie Hou, Chunli Wang, Zhijie Kang, Wenfeng Fang, Li Zhang, Eric W-F Lam, Xiang Guo, Jinsong Yan, Yixin Zeng, Mingyuan Chen, Quentin Liu

**Affiliations:** 1 Sun Yat-sen University Cancer Center; State Key Laboratory of Oncology in South China; Collaborative Innovation Center for Cancer Medicine; Guangdong Key Laboratory of Nasopharyngeal Carcinoma Diagnosis and Therapy, Guangzhou, China; 2 Institute of Cancer Stem Cell, Cancer Center, Dalian Medical University, Dalian, China; 3 Department of Hematology; Liaoning Key Laboratory of Hematopoietic Stem Cell Transplantation and Translational Medicine; Liaoning Medical Center for Hematopoietic Stem Cell Transplantation; Dalian Key Laboratory of Hematology; Diamond Bay Institute of Hematology, The Affiliated Second Hospital of Dalian Medical University, Dalian, China; 4 Department of Radiation Oncology, The Sixth Affiliated Hospital of Sun Yat-sen University, Guangzhou, China; 5 Sun Yat-sen Institute of Hematology, The Third Affiliated Hospital of Sun Yat-sen University, Guangzhou, China

**Keywords:** Cancer stem cells, Head and neck cancer

## Abstract

Application of differentiation therapy targeting cellular plasticity for the treatment of solid malignancies has been lagging. Nasopharyngeal carcinoma (NPC) is a distinctive cancer with poor differentiation and high prevalence of Epstein-Barr virus (EBV) infection. Here, we show that the expression of EBV latent protein LMP1 induces dedifferentiated and stem-like status with high plasticity through the transcriptional inhibition of CEBPA. Mechanistically, LMP1 upregulates STAT5A and recruits HDAC1/2 to the CEBPA locus to reduce its histone acetylation. HDAC inhibition restored CEBPA expression, reversing cellular dedifferentiation and stem-like status in mouse xenograft models. These findings provide a novel mechanistic epigenetic-based insight into virus-induced cellular plasticity and propose a promising concept of differentiation therapy in solid tumor by using HDAC inhibitors to target cellular plasticity.

## Introduction

Dedifferentiation processes largely enhances the cellular plasticity endowing cancer cells with dynamic adaptability and capacity to develop metastases and therapy resistance.^1^ Although it has long been appreciated that differentiation therapy revolutionizes the treatment of acute promyelocytic leukemia (APL) and dramatically improving survival, but the application of differentiation therapy targeting cellular plasticity for the treatment of solid malignancies has been lagging. Nasopharyngeal carcinoma (NPC) is a poorly differentiated form of malignancy arising from the nasopharynx epithelium.^2–4^ Over 95% NPC patients are diagnosed to have histological poorly differentiated carcinomas with aberrant cellular plasticity.^5^ This distinctive biology of NPC makes it an excellent target for differentiation therapy. We have worked on the identification of molecular mechanisms underlying the cellular plasticity of NPC, breast cancer, and leukemia.^6–10^ Specifically, we demonstrated that EZH2-mediated repression of IKKα plays an essential role in maintaining the high plasticity phenotype of NPC cells.^6^ However, the early events and initiating mechanism responsible for acquiring the aberrant plasticity of NPC remains to be determined.

Epstein-Barr virus (EBV) is a human herpesvirus that is commonly associated with multiple human malignancies.^11–14^ Previous work reported that EBV is detected ubiquitously in all undifferentiated NPC cells,^15–17^ suggesting a correlation between the undifferentiated status of NPC and EBV infection. Previous studies indicated that EBV latent membrane protein 1 (LMP1) was involved in the development of NPC progenitor cells^18–20^ and conferred these cells with the highly metastatic properties,^21–25^ suggesting a potential role of LMP1 in the aberrant plasticity phenotype of NPC. Yet, the precise activity of LMP1 in NPC development stays unclear as this membrane protein is usually expressed at low levels in a minority of NPC cells, possibly owing to proteasomal degradation.^26^ A recent immunohistochemistry (IHC) study showed that LMP1 was detected at high expression levels in 25.7% of NPC patients, which was associated with poor outcome.^27^ Hitherto, the role by which LMP1 may modulate the differentiation program remains to be clarified.

Cell state plasticity and differentiation are tightly controlled by epigenetic chromatin remodeling.^28^ A key chromatin remodeling event is the acetylation and deacetylation of histone tail lysines, which is integral to transcriptional activation and silencing.^29^ Indeed, histone deacetylases (HDACs) play a central role in cellular differentiation and cancer pathogenesis.^30–32^ HDAC inhibitors (HDACi) have been shown to be successful candidate drugs in differentiation therapy for APL, acute myeloid leukemia (AML) and cutaneous T-cell lymphoma (CTCL).^33,34^ However, whether HDACi may effectively target aberrant plasticity of solid tumor, such as poorly differentiated NPC, requires further elucidation.

Here, using conditional LMP1 expression and LMP1-inactivated cell models, we identified CEBPA as a critical restriction factor of cellular plasticity, whereas it is silenced by LMP1 in NPC progression. We further demonstrated that HDAC inhibition effectively targeted cellular plasticity *via* restoring CEBPA expression in the mice engrafted model. These findings provide novel mechanistic epigenetic-based insights into the virus-induced dedifferentiation mechanism and offer a basis for prospective clinical application using HDACi to target cellular plasticity in solid tumor differentiation therapy.

## Results

### EBV LMP1 induces dedifferentiation of NPC-derived cells and enhances tumorigenesis

**Fig. 1:**
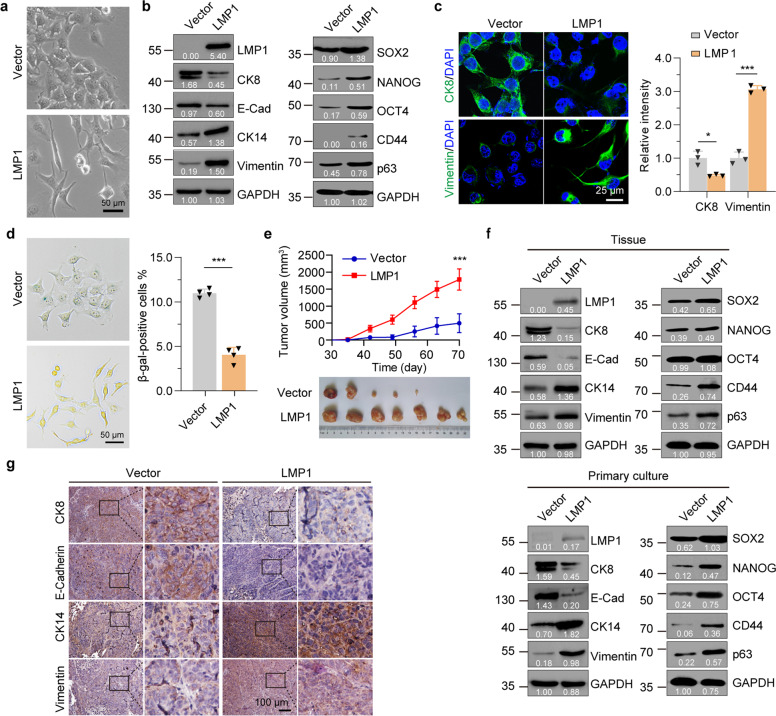
LMP1 induces dedifferentiation of NPC-derived cells and enhances tumorigenesis. **a** Phase contrast images of CNE1-TetOn-Vector (Vector) and CNE1-TetOn-LMP1 (LMP1) cells treated with 100 ng/ml Dox for 48 hours. **b**, **c** Immunofluorescence staining with differentiation markers in CNE1-TetOn-Vector and CNE1-TetOn-LMP1 cells treated with 100 ng/ml Dox for 48 hours. **d** SA-β-gal staining in CNE1-TetOn-Vector and CNE1-TetOn-LMP1 cells treated with 100 ng/ml Dox for 48 hours. **e** CNE1-TetOn-Vector and CNE1-TetOn-LMP1 cells were injected into nude mice subcutaneously with continuous Dox administration and tumor volume was determined. **f** Tumor tissue or primary cultured tumor cells obtained by isolating cells from trypsinized tumor tissue were subjected to western blot with the indicated antibodies. **g** Immunohistochemistry with differentiation markers in tumor from mice with Dox administration. Representative immunohistochemistry images are shown. Statistics (**c**–**e**), significance: *P < 0.05, ***P < 0.001; two-tailed Student’s *t*-tests

To determine whether LMP1 induces dedifferentiation of NPC-derived CNE1 and HNE2 cells, we established a doxycycline (Dox) inducible (Tet-on) LMP1 lentiviral expression system in these cells (named as “CNE1/HNE2-TetOn-LMP1”, abbreviated as “LMP1”) and the empty Vector control cells (named as “CNE1/HNE2-TetOn-Vector”, abbreviated as “Vector”, Supplementary Fig. 1a). Treatment of LMP1 cells with Dox resulted in LMP1 expression in a dose-dependent manner in both CNE1 and HNE2 cells (Supplementary Fig. 1b, c). To mimic the physiological protein level, we select 100 ng/ml of Dox to induce LMP1 expression for the following studies. The induction of LMP1 led to the dedifferentiation of CNE1 cells, which changed markedly from an epithelial to a fibroblast-like morphology and converted to loosely connected cells (Fig. 1a and Supplementary Fig. 1d). Concomitantly, the expression levels of NPC differentiation markers (eg. E-Cadherin and CK8) decreased, whereas the undifferentiated (eg. Vimentin and CK14) and stem-like (SOX2, NANOG, OCT4, CD44, and p63) markers increased after treatment of CNE1-TetOn-LMP1 cells with Dox (Fig. 1b, c). Similar results were observed in the moderate differentiated HNE2-TetOn-LMP1 cells (Supplementary Fig. 2a–d). Furthermore, knockdown of LMP1 in C666-1 cells which inherently harbors the EBV genome or in HK1-EBV cells which are infected by EBV, increased the expression of differentiation markers, and decreased the expression of undifferentiated and stem-like markers, suggesting there is a reversion of the undifferentiated phenotype (Supplementary Fig. 3a, c, d). Both cell proliferation and colony formation assays showed that expression of LMP1 increased cell growth and clonogenicity in well and moderate differentiated CNE1 and HNE2 cells (Supplementary Fig. 2e, f), while knockdown of LMP1 decreased cell growth in C666-1 cells and HK1-EBV cells (Supplementary Fig. 3b, e). In addition, induction of LMP1 significantly increased the ratio of Ki67 cells and decreased the population of senescence-associated (SA) β-gal-positive cells (Supplementary Fig. 2g and Fig. 1d), suggesting that LMP1 can override the senescence program.

To address whether LMP1 induces dedifferentiation of CNE1 cells in vivo, we subcutaneously injected CNE1-TetOn-Vector and CNE1-TetOn-LMP1 cells into mice followed by Dox administration. CNE1-TetOn-LMP1 cells showed significant advantages in tumorigenesis upon continuous Dox administration (Fig. 1e). The corresponding tumor tissues were also isolated for analysis of the differentiated status. Consistent with the in vitro findings, differentiation markers were downregulated, while undifferentiated and stem-like markers were upregulated in LMP1-positive tumor tissues (Fig. 1f, upper panel). To exclude the normal cells from the tumor tissues in the study, tumor tissues were digested and then cultured in the selection medium with puromycin for 24 hours. Similar dedifferentiated effects were observed in the xenograft cells after selection (Fig. 1f, lower panel). Immunohistochemical staining for differentiation-related markers of primary tumors derived from Vector and LMP1 cells confirmed the phenotype of dedifferentiation in CNE1 cells after LMP1 induction (Fig. 1g). Together these data demonstrated that expression of LMP1 in NPC-derived cells induces dedifferentiation in vitro and in vivo.

### LMP1 signaling inhibits CEBPA transcription in NPC-derived cells

**Fig. 2:**
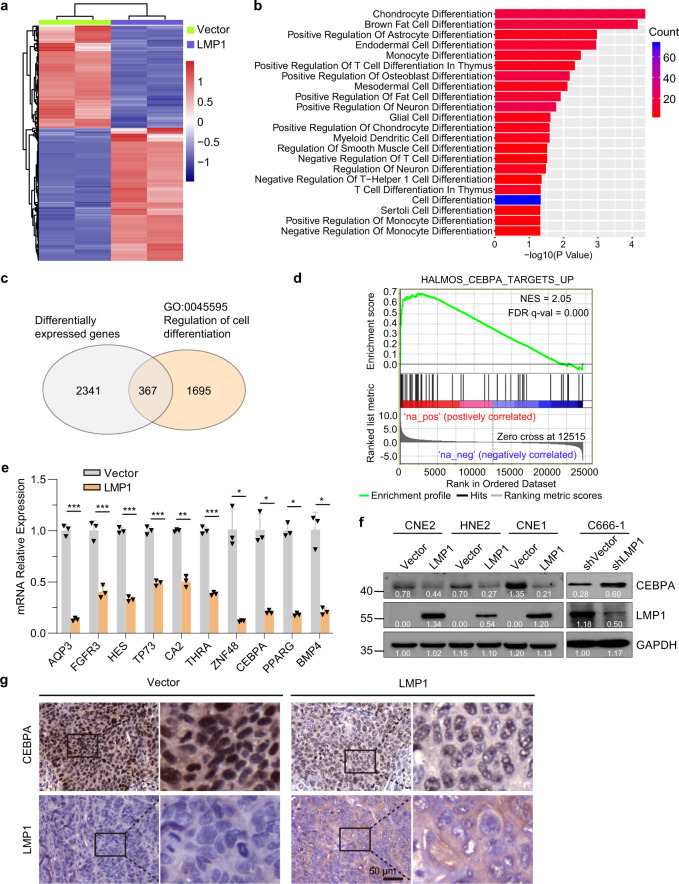
LMP1 signaling inhibits CEBPA transcription in the NPC-derived cells. **a** Heatmap analysis showing significantly differentially expressed genes (fold change >2) in the CNE1-TetOn-LMP1 (LMP1) cells compared to the CNE1-TetOn-Vector (Vector) cells after 100 ng/ml Dox treatment for 48 hours. **b** Functional categorization of differentially expressed genes analyzed for GO biological processes. **c** In all, 367 differentially expressed genes are cell differentiation-related genes, analyzed by g: Profiler. **d** GSEA analysis indicates enriched expression of direct CEBPA transcriptional targets upon induction of LMP1. **e** Differentially expressed genes in GO term “regulating of cell differentiation”, including CEBPA, were validated by RT-qPCR. **f** CNE1/HNE2/CNE2-TetOn-LMP1 and CNE1/HNE2/CNE2-TetOn-Vector cells were treated by 100 ng/ml Dox, or knockdown of LMP1 in C666-1 cells for 48 hours, and then the expression of CEBPA was measured by immunoblotting. **g** IHC staining of CEBPA expression in sections from xenografts of CNE1-TetOn-LMP1. Statistics, significance: *P < 0.05, **P < 0.01, ***P < 0.001; two-tailed Student’s *t-*tests

We next performed expression profiling using RNA-sequencing to elucidate the mechanism by which LMP1 induces dedifferentiation of NPC-derived CNE1 cells. To this end, Vector and LMP1 cells were treated with Dox for 48 hours before isolation for RNA-sequencing analysis. We then analyzed the global gene expression changes upon induction of LMP1 in CNE1 cells. As expected, expression of LMP1 led to alterations of the global transcription profile, which include the upregulation of 1680 genes and downregulation of 1180 genes (Fig. 2a). Gene ontology analysis revealed that differentiation-related biology processes were regulated by LMP1 (Fig. 2b). Of the differentially expressed genes, 367 genes were cell differentiation-related genes (Fig. 2c). Gene set enrichment analysis (GSEA) revealed that CEBPA target gene signatures are distinctly enriched in the gene expression profiles of the LMP1-induction group (Fig. 2d). Consistently, suppression of CEBPA and other differentiation-related genes was observed in the LMP1-positive CNE1 cells (Fig. 2e). In agreement, immunofluorescence staining showed that the nuclear CEBPA was decreased in LMP1-positive CNE1 and HNE2 cells (Supplementary Fig. 4a, b). In addition, the protein and mRNA levels of CEBPA were generally decreased by induction of LMP1 in CNE2, HNE2 and CNE1 cell lines with distinct differentiation gradings (Fig. 2f and Supplementary Fig. 4c), while knockdown of LMP1 increased CEBPA expression in C666-1 cells (Fig. 2f).

We next determined the expression levels of CEBPA in tumor xenografts with or without LMP1 expression. The results again showed that the mRNA levels of CEBPA were decreased in the tumor cells with LMP1 expression (Supplementary Fig. 4d). Immunohistochemical analysis revealed decreased CEBPA staining in tumor sections with LMP1 expression, compared with the control tumor sections (Fig. 2g). These data suggested that the expression of LMP1 regulates differentiation signaling in NPC-derived cells and suppresses the expression of differentiation-inducing transcription factor CEBPA.

### CEBPA drives cellular differentiation of NPC-derived cells

**Fig. 3:**
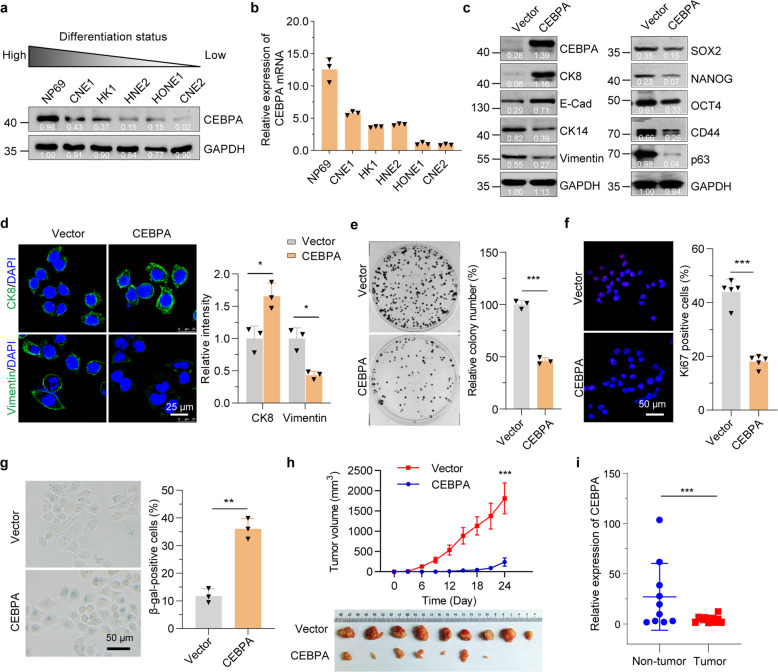
CEBPA drives cellular differentiation of NPC-derived cells. **a** Protein levels of CEBPA in various NPC-derived cell lines with different differentiation status. **b** RT-qPCR analysis of relative CEBPA mRNA levels in various NPC-derived cell lines with different differentiation status. **c** Western blot with differentiation and stem-like markers in control (Vector) and CEBPA-overexpressed (CEBPA) CNE2 cells. **d** Immunofluorescence analysis with differentiation markers in control and CEBPA-overexpressed CNE2 cells. **e** Colony formation in control and CEBPA-overexpressed CNE2 cells. **f** Immunofluorescence staining for Ki67 in control and CEBPA-overexpressed CNE2 cells. **g** SA-β-gal staining in control and CEBPA-overexpressed CNE2 cells. **h** CEBPA-overexpressed and control CNE2 cells were injected into nude mice subcutaneously, and tumor volume was determined. **i** RT-qPCR analysis of relative CEBPA mRNA levels in patients’ tumor tissues non-tumor nasopharyngeal tissues. Statistics (**d**–**i**), significance: *P < 0.05, **P < 0.01, ***P < 0.001; two-tailed Student’s *t*-tests

We next assessed whether the expression of CEBPA is correlated to the differentiated status of NPC-derived cells. Protein levels of CEBPA were examined in several tumor and normal nasopharyngeal epithelial cell lines of well differentiated to poorly differentiated status. In concordance, CEBPA was highly expressed in the well-differentiated cell lines, while expression remained low in poorly differentiated cell lines (Fig. 3a). Similar results were observed for the transcription levels of CEBPA (Fig. 3b).

We then asked if CEBPA expression is responsible for the differentiation of NPC cells. As shown in Fig. 3c and Supplementary Fig. 5a, overexpression of CEBPA in undifferentiated CNE2 cells and C666-1 cells indeed induced their differentiation. Immunostaining of differentiation markers further confirmed the differentiation phenotypes (Fig. 3d). To corroborate further the role of CEBPA in NPC, we utilized functional assays to evaluate the effects of CEBPA ectopic expression. Compared to control CNE2 cells, overexpression of CEBPA significantly decreased the colony formation ability of CNE2 cells and reduced the ratio of the proliferation-associated Ki67-positive cells (Fig. 3e, f). Similarly, overexpression of CEBPA significantly decreased the proliferation of C666-1 cells (Supplementary Fig. 5b). In addition, ectopic expression of CEBPA largely increased the proportion of senescence-associated (SA) β-gal-positive cells (Fig. 3g).

We next assessed the impact of ectopic CEBPA expression on the tumorigenesis. CNE2 cells expressing control or CEBPA were subcutaneously injected into nude mice and primary tumor growth assessed over time. We found CNE2 cells expressing CEBPA showed a significant reduction in tumor growth compared to control cells (Fig. 3h). Importantly, the expression of CEBPA in NPC patients’ tissues was significantly decreased comparing to the non-tumor nasopharyngeal tissues (Fig. 3i). Together, these findings indicated that CEBPA is a key regulator of induced differentiation in NPC cells.

### CEBPA restoration induces differentiation and overcomes cellular plasticity in NPC-derived cells

**Fig. 4:**
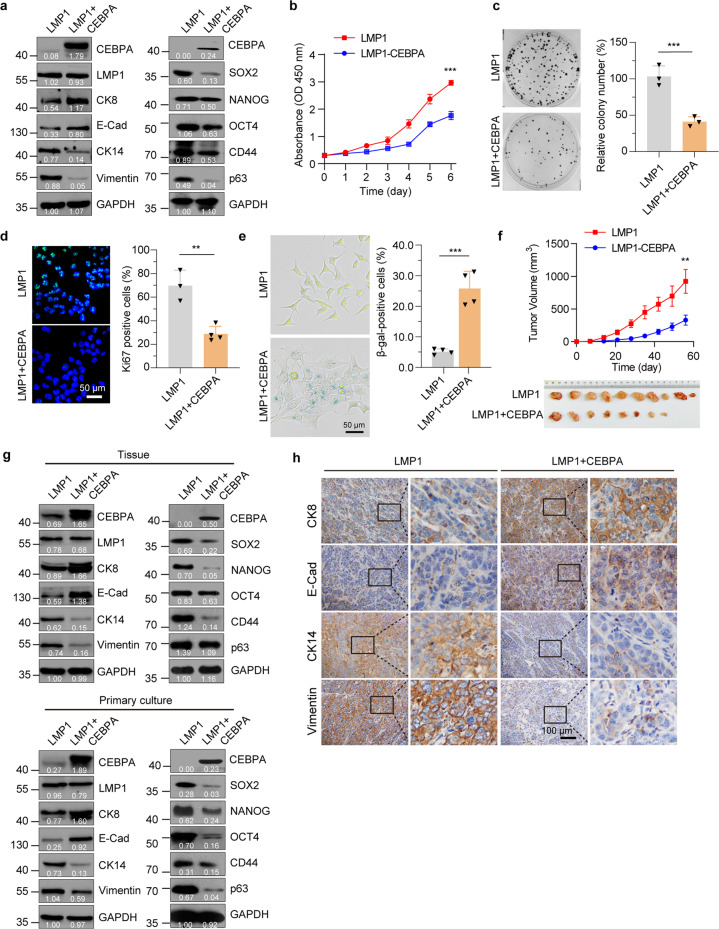
CEBPA overcomes LMP1-induced dedifferentiation in NPC cells. **a** Western blot analysis with differentiation and stem-like markers in CNE1-TetOn-LMP1 and CEBPA-overexpressed CNE1-TetOn-LMP1 cells by 100 ng/ml Dox treatment for 48 hours. **b** Proliferation ability of CNE1-TetOn-LMP1 and CEBPA-overexpressed CNE1-TetOn-LMP1 cells was determined by CCK8 assay. **c** Colony formation in CNE1-TetOn-LMP1 and CEBPA-overexpressed CNE1-TetOn-LMP1 cells. **d** Immunofluorescence staining for Ki67 in CNE1-TetOn-LMP1 and CEBPA-overexpressed CNE1-TetOn-LMP1 cells. **e** SA-β-gal staining in CNE1-TetOn-LMP1 and CEBPA-overexpressed CNE1-TetOn-LMP1 cells. **f** CNE1-TetOn-LMP1 and CEBPA-overexpressed CNE1-TetOn-LMP1 cells were injected into nude mice subcutaneously. After continuous Dox administration, tumor volume was determined. **g** CEBPA, differentiation, and stem-like markers were analyzed by immunoblot in tumor tissue and primary cultured tumor cells. **h** IHC staining of differentiation markers in CNE1-TetOn-LMP1 and CEBPA-overexpressed CNE1-TetOn-LMP1 tumor tissues. Representative images are shown. Statistics (**b**–**f**), significance: **P < 0.01, ***P < 0.001; two-tailed Student’s *t*-test

To ascertain whether restored expression of CEBPA can induce differentiation to overcome cellular plasticity in the poorly differentiated LMP1-expressing NPC-derived cells, we generated a CNE1 cell line that co-expressing LMP1 and CEBPA after the Dox induction (CNE1-TetOn-LMP1-IRES-CEBPA, named as LMP1 + CEBPA). We first studied the differentiation markers to determine whether CEBPA expression itself is sufficient to induce differentiation. Indeed, comparing to the CNE1-TetOn-LMP1 cells, restoration of CEBPA expression in CNE1-TetOn-LMP1 cells was sufficient to induce differentiation as indicated by the upregulation of differentiation markers (E-Cadherin, CK8) and downregulation of undifferentiated (Vimentin, CK14) and stem-like (SOX2, NANOG, OCT4, CD44, and p63) markers (Fig. 4a). Moreover, induction of CEBPA significantly inhibited the proliferation and clonogenic potential of CNE1-TetOn-LMP1 cells (Fig. 4b, c). Meanwhile, the ratio of Ki67-positive cells was decreased and the proportion of senescence-associated (SA) β-gal-positive cells was increased after CEBPA reconstitution (Fig. 4d, e).

To assess whether CEBPA suppresses LMP1-mediated tumorigenesis in vivo, we injected CNE1-TetOn-LMP1 cells and CNE1-TetOn-LMP1-IRES-CEBPA cells subcutaneously into immunocompromised mice. Upon continuous Dox administration, CNE1-TetOn-LMP1-IRES-CEBPA cells gave rise to slower tumor growth and reduced tumor sizes compared with CNE1-TetOn-LMP1 cells (Fig. 4f). To examine the differentiation status of tumor tissues, we dissociated tumor tissues and investigated the expression of the differentiation markers. Consistent with the in vitro results, restoration of CEBPA increased the expression of differentiation markers and decreased the expression of undifferentiated and stem-like markers in these tissue samples (Fig. 4g, upper panel). To eliminate the normal cells from the tumor tissues, tumor tissues were digested and then cultured in the selection medium with puromycin for 24 hours. Similar results were again observed in the xenograft cells after puromycin selection (Fig. 4g, lower panel). Immunohistochemical staining for differentiation-related markers of primary tumors derived from CNE1-TetOn-LMP1 and CNE1-TetOn-LMP1-IRES-CEBPA cells confirmed the differentiation of tumor tissues after CEBPA restoration (Fig. 4h). Collectively, these findings demonstrated that CEBPA restoration can effectively impair cellular plasticity to induce redifferentiation in LMP1-induced dedifferentiated NPC-derived cells.

### Histone deacetylation is required for LMP1-mediated CEBPA repression

Silencing tumor suppressor CEBPA via promoter hypermethylation has been reported in a number of human cancers.^35,36^ To explore whether the expression of CEBPA is suppressed by LMP1-mediated hypermethylation, we treated CNE1-TetOn-Vector and CNE1-TetOn-LMP1 cells with five dose series of the DNA-demethylating agent 5-aza-2′-deoxycytidine (Decitabine, DAC). The protein level of CEBPA was decreased in the CNE1-TetOn-LMP1 group, and DAC treatment showed no effects on the recovery of CEBPA expression (Supplementary Fig. 6a). We also performed methylation-specific PCR (MSP) analysis and bisulfite sequencing PCR (BSP) to assess the methylation status of the *CEBPA* promoter. A similar pattern of DNA methylation occurred in CNE1-TetOn-Vector and CNE1-TetOn-LMP1 cells without Dox administration (Supplementary Fig. 6b, c).

LMP1 induction can trigger various downstream oncogenic signaling cascades, including the JAK/STAT, NF-κB, MAPK, and PI3K/Akt pathways.^37^ To explore if the CEBPA silencing by LMP1 is mediated through these classical signaling cascades, we treated the CNE1-TetOn-Vector and CNE1-TetOn-LMP1 cells with JNK inhibitor SP600125, NF-κB inhibitor BAY11-7028, MEK inhibitor PD98059, and PI3K inhibitor wortmannin, respectively. All these inhibitor treatments failed to restore the expression of CEBPA in CNE1-TetOn-LMP1 cells (Supplementary Fig. 6d–g). Considering that LMP1 is well known to promote NF-κB signaling and CEBPA is a direct transcriptional target of NF-κB,^38^ we therefore further confirmed the higher nuclear translocation of p65 and p50 in LMP1-overexpressed cells (Supplementary Fig. 6h). The NF-κB canonical targets CXCL2, CXCL1, IL2β, IL8, and IL6 were also activated, while CEBPA was suppressed in LMP1-overexpressed cells (Supplementary Fig. 6i). We further attenuated NF-κB activity via knockdown p65, and found *CEBPA* promoter activity was still inhibited by LMP1 overexpression (Supplementary Fig. 6j, k). These results indicate that NF-κB activation does not activate CEBPA at the transcriptional level in NPC. In addition, we found that the mRNA stability of CEBPA was not affected by LMP1 induction in CNE1 cells (Supplementary Fig. 6l).

**Fig. 5:**
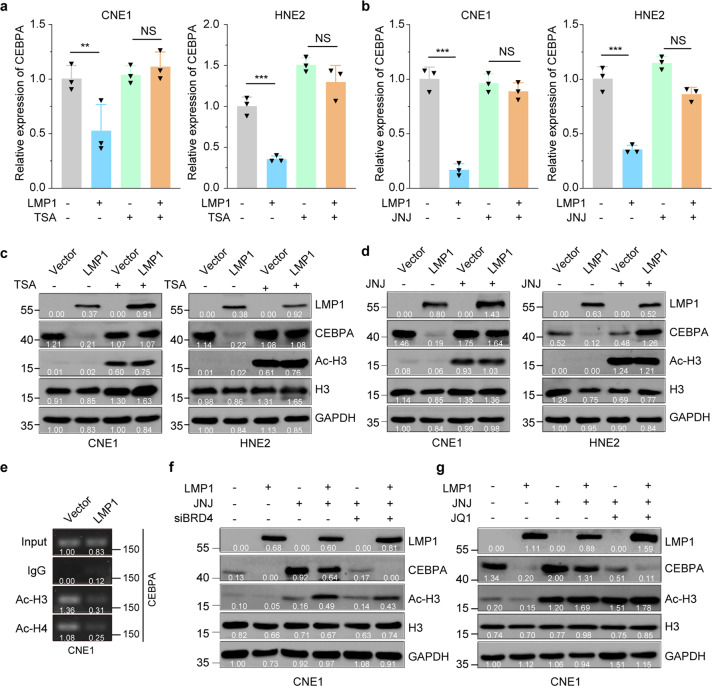
Histone deacetylation is required for LMP1-induced CEBPA repression. **a**, **b** mRNA levels of CEBPA were determined by RT-qPCR. CNE1/HNE2-TetOn-Vector and CNE1/HNE2-TetOn-LMP1 cells were pretreated with 100 ng/ml Dox for 24 hours then followed by 1 µM TSA or 200 nM JNJ treatment for further 24 hours. **c**, **d** Protein levels of CEBPA were determined by immunoblotting. CNE1/HNE2-TetOn-Vector and CNE1/HNE2-TetOn-LMP1 cells were pretreated with 100 ng/ml Dox for 24 hours then followed by 1 µM TSA or 200 nM JNJ treatment for further 24 hours. **e** CNE1-TetOn-Vector and CNE1-TetOn-LMP1 cells were treated with 100 ng/ml Dox for 48 hours. ChIP assays were performed using anti-acetylated histone H3 and anti-acetylated histone H4 antibodies. **f**, **g** Protein levels of CEBPA were determined by immunoblotting. Knockdown of BRD4 by siRNA or 5 µM BRD4 inhibitor treatment in CNE1-TetOn-Vector and CNE1-TetOn-LMP1 cells then LMP1 expression were induced by 100 ng/ml Dox for 24 hours and exposed to 200 nM JNJ for further 24 hours. Statistics (**a**, **b**), significance: **P < 0.01, ***P < 0.001; two-tailed Student’s *t*-tests

As various HDAC complexes have been identified to be linked to the repression of transcription,^39^ we next investigated whether HDACs are involved in LMP1-mediated CEBPA suppression. CNE1-TetOn-Vector and CNE1-TetOn-LMP1 cells were treated with the histone deacetylases inhibitor (HDACi) trichostatin A (TSA) or Quisinostat (JNJ‑26481585, abbreviated to JNJ), a novel second‑generation HDACi. We found that both TSA and JNJ could recover the mRNA level of CEBPA after the LMP1 induction in both CNE1 and HNE2 cells (Fig. 5a, b). Indeed, TSA treatment increased the level of histone 3 acetylation in both CNE1-TetOn-Vector and CNE1-TetOn-LMP1 cells and restored the CEBPA expression in CNE1-TetOn-LMP1 cells. Consistent results were also observed in HNE2-TetOn-Vector and HNE2-TetOn-LMP1 cells following TSA treatment (Fig. 5c). Moreover, similar results were also obtained with the JNJ treatment in the two cell lines (Fig. 5d). These findings suggested the mRNA level of CEBPA is suppressed by LMP1-induced histone deacetylation.

We further assessed the acetylation levels of histones H3 and H4 at the *CEBPA* promoter loci, which are associated with transcriptional activation. LMP1 induction in CNE1 cells significantly decreased the H3 and H4 acetylation at the *CEBPA* promoter (Fig. 5e). Histone acetylation modifications need to be recognized by the “acetyl-lysine reader” before the subsequent transcriptional events, and Bromodomain Containing 4 (BRD4) is the most thoroughly characterized acetyl-lysine reader.^40^ To confirm whether HDACi induced CEBPA reexpression in LMP1-expressing CNE1-TetOn-LMP1 cells was caused by increasing histone acetylation mediated by BRD4 at the *CEBPA* promoter locus, we utilized BRD4 siRNA to deplete BRD4 expression in CNE1-TetOn-Vector and CNE1-TetOn-LMP1 cells before treatment with JNJ. The acetylation level of histone H3 was remarkably increased in both BRD4 knockdown and control cells after JNJ treatment. However, CEBPA expression was only restored in the controls but not the BRD4 knockdown cells (Fig. 5f and Supplementary Fig. 6m). These results were further verified by using the compound JQ1, a pharmacological inhibitor of BRD4. Specifically, JQ1 treatment significantly restricted the JNJ-induced CEBPA transcriptional restoration in the LMP1-expressing NPC-derived cells (Fig. 5g and Supplementary Fig. 6n). Collectively these results proposed a model in which JNJ treatment increases the histone acetylation at the promoter region of CEBPA gene, resulting in the recruitment of BRD4 for the restoration of CEBPA expression in the LMP1-expressing NPC-derived cells.

### Concurrent inhibition of HDAC1 and HDAC2 reverses LMP1-mediated CEBPA repression

**Fig. 6:**
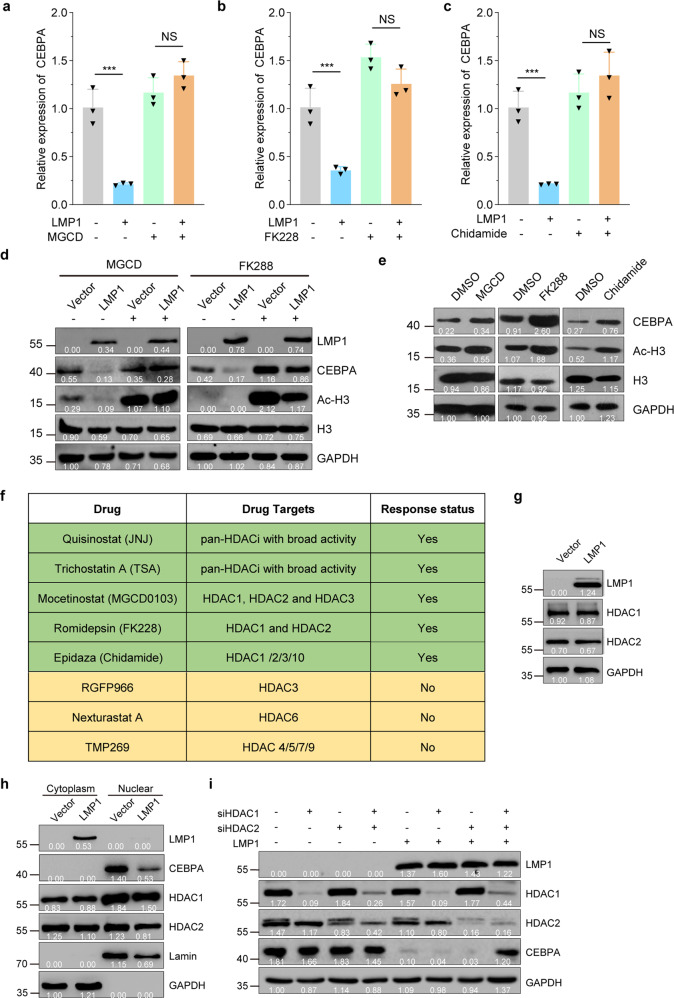
Concurrent inhibition of HDAC1 and HDAC2 reverses LMP1-mediated CEBPA repression. **a**–**c** CNE1-TetOn-LMP1 andCNE1-TetOn-Vector cells were treated by 100 ng/ml Dox for 24 hours, and then exposed to 10 µM MGCD, 10 nM FK228, and 1 µM Chidamide for further 24 hours, respectively. Then mRNA levels of CEBPA were determined by RT-qPCR. **d** CNE1-TetOn-LMP1 and CNE1-TetOn-Vector cells were treated by 100 ng/ml Dox, and then exposed to 10 µM MGCD and 10 nM FK228 for further 24 hours, respectively. Then protein levels of CEBPA were determined by immunoblotting. **e** C666-1 cells were exposed to 10 µM MGCD, 10 nM FK228, and 1 µM Chidamide for 24 hours, respectively. Then protein levels of CEBPA were determined by immunoblotting. **f** Summary of mRNA level of CEBPA restoration by HDACi treatment in 100 ng/ml Dox induced CNE1-TetOn-LMP1 cells. **g** Western blot assays of HDAC1 and HDAC2 in Dox-induced CNE1-TetOn-LMP1 and CNE1-TetOn-Vector cells. **h** The cytoplasmic and nuclear protein lysates representing an equal number of CNE1-TetOn-LMP1 and CNE1-TetOn-Vector cells subjected to immunoblot. **i** CNE1-TetOn-LMP1 and CNE1-TetOn-Vector cells were transfected with siRNA of HDAC1 or HDAC2. Expression levels of CEBPA were examined by immunoblotting. Statistics (**a**–**c**), significance: ***P < 0.001, NS no significance; two-tailed Student’s *t*-tests

Human HDACs consist of more than 10 deacetylases. To determine which HDAC or HDACs were required for LMP1-induced CEBPA transcriptional suppression in NPC-derived cells, we treated CNE1-TetOn-Vector and CNE1-TetOn-LMP1 with various HDACi targeting different types of HDACs after LMP1 induction. The results showed that Mocetinostat (MGCD0103), Romidepsin (FK228), and Epidaza (Chidamide) treatment effectively restored CEBPA mRNA levels in CNE1-TetOn-LMP1 cells after the LMP1 induction (Fig. 6a–c). On the other hand, RGFP966, Nexturastat A, or TMP269 failed to restore CEBPA mRNA expression in CNE1-TetOn-LMP1 cells after LMP1 induction (Supplementary Fig. 7a–c).

We next examined whether CEBPA protein levels are also restored by various HDACi treatments in CNE1-TetOn-LMP1 cells after the LMP1 induction. Consistently, MGCD0103 or FK228 treatment restored the protein expression level of CEBPA in CNE1-TetOn-LMP1 cells after the LMP1 induction (Fig. 6d). We further showed that MGCD0130, FK228, and Chidamide treatment restored the protein expression level of CEBPA in C666-1 cells (Fig. 6e). Likewise, RGFP966, Nexturastat and TMP269 failed to restore protein levels of CEBPA in CNE1-TetOn-LMP1 cells after the LMP1 induction (Supplementary Fig. 7d–f). Collectively, these results, summarized in Fig. 6f, suggested that HDAC1 and HDAC2 simultaneous inhibition are required for the restoration of CEBPA in the CNE1-TetOn-LMP1 cells after the LMP1 induction, or in C666-1 cells. It is possible that LMP1-mediated CEBPA suppression through modulating the activity of HDAC1 and HDAC2. Interestingly, we found LMP1 induction did not alter the expression of HDAC1 or HDAC2 (Fig. 6g). In addition, the nuclear and cytosolic distributions of HDAC1 and HDAC2 have similar patterns in Dox-treated CNE1-TetOn-Vector and CNE1-TetOn-LMP1 cells (Fig. 6h).

To determine whether HDAC1 and HDAC2 are indeed responsible for the LMP1-mediated CEBPA suppression, we depleted HDAC1 and HDAC2 individually and simultaneously by siRNAs in Dox-treated CNE1-TetOn-Vector and CNE1-TetOn-LMP1 cells. Intriguingly, only simultaneously, but not individual, knockdown of HDAC1 and HDAC2 blocked the LMP1-mediated CEBPA suppression in Dox-treated CNE1-TetOn-Vector and CNE1-TetOn-LMP1 cells (Fig. 6i), suggesting that both HDAC1 and HDAC2 are required for LMP1-mediated CEBPA suppression.

### STAT5A recruits HDAC1/2 to CEBPA gene locus

**Fig. 7:**
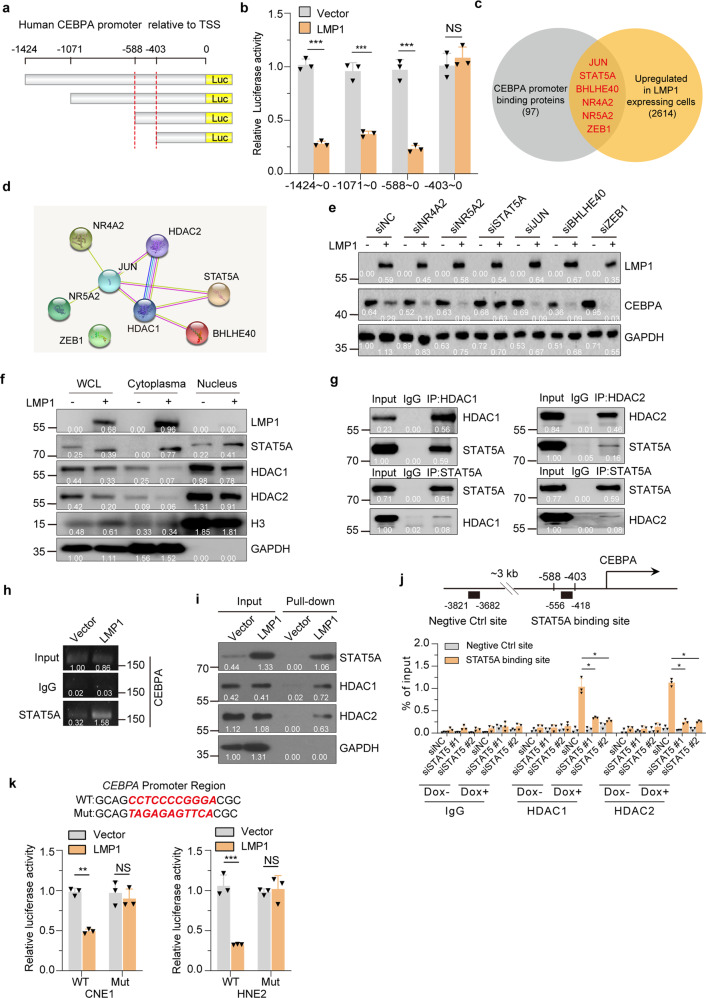
STAT5A recruits HDAC1/2 to CEBPA Gene Locus. **a**, **b** Truncated CEBPA promoter luciferase activity in CNE1-TetOn-LMP1 and CNE1-TetOn-Vector cells were detected by dual-luciferase reporter assays. **c** Prediction of known transcription factor binding in the −588 to −403 region. **d** STRING analysis of known transcription factor binding in the −588 to −403 region and HDAC1/2. Image shows the physical and functional interactions. **e** CNE1-TetOn-LMP1 and CNE1-TetOn-Vector cells were transfected with siRNAs of indicated TFs. Then expression levels of CEBPA were examined by immunoblotting. **f** Western blot analysis of the expression and localization of STAT5A, HDAC1, and HDAC2 in CNE1-TetOn-LMP1 and CNE1-TetOn-Vector cells. **g** Western blot analysis of the co-immunoprecipitates between endogenous HDAC1 and STAT5A, and HDAC2 and STAT5A. **h** CNE1-TetOn-LMP1 and CNE1-TetOn-Vector cells were treated with 100 ng/ml Dox for 48 hours. Cells were collected and ChIP assays were performed using anti-STAT5A. ChIP signals were detected by PCR using primers for the CEBPA promoter region. **i** Biotinylated CEBPA promoter pull-down assay was performed. The associated proteins were subjected to immunoblot analysis with indicated antibodies. **j** STAT5A was knockdown by siRNA in CNE1-TetOn-LMP1 and CNE1-TetOn-Vector cells. Cells were collected and ChIP assays were performed using anti-HDAC1 and anti-HDAC2. ChIP signals were detected by qRT-PCR using primers for the CEBPA promoter region. **k** Wild-type or mutant CEBPA promoter luciferase activity in CNE1/HNE2-TetOn-LMP1 and CNE1/HNE2-TetOn-Vector cells was detected by dual-luciferase reporter assays. Statistics (**b**, **j** and **k**), significance: *P < 0.05, **P < 0.01, ***P < 0.001, NS: no significance; two-tailed Student’s *t-*tests

To interrogate further the mechanism by which LMP1 induction suppresses CEBPA expression via HDAC1 and HDAC2, we generated a series of *CEBPA* promoter truncations. Luciferase reporter assay, showed that LMP1 lost the ability to repress the transcription of CEBPA when the promoter was truncated from −588 to −403 bp (Fig. 7a, b). Similar results were obtained in HNE2-TetOn-Vector and HNE2-TetOn-LMP1 cells (Supplementary Fig. 8a). The −588 to −403 region in *CEBPA* promoter contained consensus binding sequences for transcription factors that can potentially recruit HDAC1/2. Of which, six of the transcription factors were upregulated after LMP1 induction (Fig. 7c). These six predicted transcription factors together with HDAC1 and HDAC2 were then subjected to STRING analysis, and the result indicated that HDAC1 and HDAC2 can interact with each other and form complexes with JUN, BHLHE40 and STAT5A (Fig. 7d).

To identify the transcription factors involved in repressing CEBPA transcription in LMP1-expressing cells, we screened the six predicted transcription factors using siRNAs in CNE1-TetOn-Vector and CNE1-TetOn-LMP1 cells. Knockdown efficiencies of siRNAs were validated by RT-qPCR in CNE1 cells (Supplementary Fig. 8b). Our results revealed that only depletion of STAT5A blocked the LMP1-induced CEBPA repression and restored the CEBPA expression in CNE1-TetOn-LMP1 cells (Fig. 7e and Supplementary Fig. 8c).

To further ascertain the role of STAT5A in LMP1-induced CEBPA repression, we first examined the expression of STAT5A. LMP1 expression induced STAT5A promoter activation and its nuclear translocation, whereas LMP1 expression did not increase the protein levels of HDAC1 and HDAC2 in the nucleus (Supplementary Fig. 8d–e and Fig. 7f). Co-immunoprecipitation assays demonstrated that the LMP1-driven nuclear STAT5A bound to HDAC1 and HDAC2 (Fig. 7g). ChIP analysis demonstrated that higher levels of STAT5A were recruited to the endogenous *CEBPA* promoter in the CNE1-TetOn-LMP1 cells when compared to the CNE1-TetOn-Vector cells after Dox treatment (Fig. 7h). In further agreement, the oligonucleotide pull-down assays of biotin-labeled *CEBPA* promoter probes (−588 to −403) showed that the STAT5A-HDAC1-HDAC2 complex can indeed bind to the *CEBPA* promoter region (−588 to −403) in LMP1-expressing cells (Fig. 7i). ChIP RT-qPCR assays demonstrated that knockdown of STAT5A suppressed the binding of HDAC1 and HDAC2 to *CEBPA* promoter (Fig. 7j). Moreover, the ability of LMP1 to repress of *CEBPA* promoter activity in CNE1-TetOn-LMP1 cells was lost when the STAT5A-binding site was mutated in the luciferase reporter assays (Fig. 7k).

Collectively, these results revealed that LMP1 induces STAT5A expression and DNA binding to recruit HDAC1 and HDAC2 to the *CEBPA* promoter to mediate transcriptional repression in the NPC-derived cells.

### HDAC inhibition induces differentiation of LMP1-positive NPC-derived cells

**Fig. 8:**
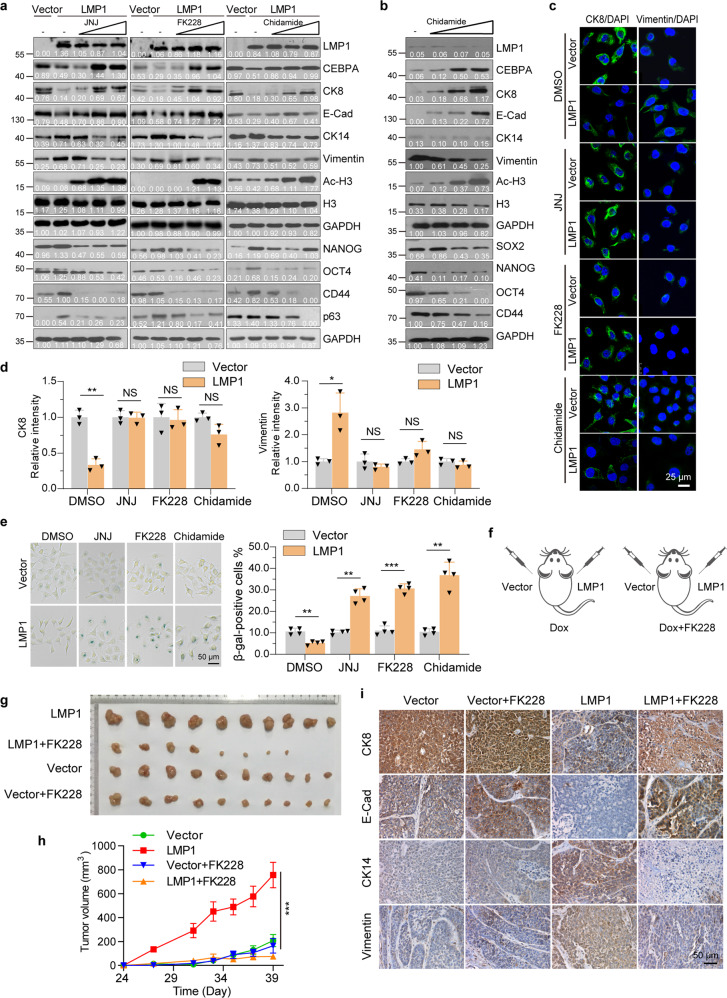
HDAC inhibition induces differentiation of LMP1-positive NPC-derived cells. **a** CNE1-TetOn-LMP1 and CNE1-TetOn-Vector cells were treated by 100 ng/ml Dox for 24 hours, and then exposed to JNJ (0, 2, 20, and 200 nM), FK228 (0, 0.5, 5, and 50 nM), or Chidamide (0, 0.5, 1, and 3 μM) for 24 hours. Expression of CEBPA, differentiation and stem-like markers were examined by immunoblotting. **b** C666-1 cells were treated with Chidamide (0, 0.5, 1, and 3 μM) for 48 hours. Expression of CEBPA, differentiation and stem-like markers were examined by immunoblotting. **c**, **d** CNE1-TetOn-LMP1 and CNE1-TetOn-Vector cells were treated by 100 ng/ml Dox for 24 hours, and then exposed to 200 nM JNJ, 10 nM FK228 or 1 μM Chidamide for 24 hours and immunofluorescence staining was performed with indicated antibodies. **e** SA-β-gal staining was performed and the β-gal-positive staining cells were counted. **f** Experimental setup for FK228 treatment. CNE1-TetOn-LMP1 and CNE1-TetOn-Vector cells were injected into nude mice subcutaneously with continuous Dox administration. Mice were treated with FK228 by intraperitoneal perfusion from day 27 (120 μg/kg, every two days). **g**, **h** The images of dissected tumors at the endpoint of the experiment were shown and growth curve was plotted by measuring the relative tumor volume at indicated day. **i** Immunohistochemistry was performed in tumors from **g** for differentiation markers. Representative images were shown. Statistics (**d**–**e**, and **h**), significance: *P < 0.05, **P < 0.01, ***P < 0.001; two-tailed Student’s *t-*tests (**d**, **e**); one-way ANOVA with Bonferroni correction (**h**)

We next investigated if LMP1-induced dedifferentiated NPC-derived cells are sensitized to treatment with HDACi Romidepsin (FK228, which targets both HDAC1 and HDAC2, an US FDA-approved drug for the treatment of cutaneous and peripheral T-cell lymphomas^41^) and Epidaza (Chidamide, which targets HDAC1, HDAC2, HDAC3, as well as Class IIb HDAC10,^42^ a Chinese CFDA-approved drug for treatment of relapsed or refractory peripheral T-cell lymphoma^43^). To this end, we treated CNE1-TetOn-LMP1 cells with various doses of the pan HDACi JNJ, FK228, or Chidamide. Western blot analysis showed that treatment with JNJ, FK228, or Chidamide efficiently restored the CEBPA expression via increasing the histone acetylation levels, and induced the cellular differentiation of CNE1-TetOn-LMP1 cells (Fig. 8a). Consistently, C666-1 cells, HNE2-TetOn-LMP1 cells, and HK1-EBV cells also displayed similar effects upon JNJ, FK228, or Chidamide treatment (Fig. 8b and Supplementary Fig. 9a,b). The differentiation/proliferative status of cells was further confirmed by the immunofluorescence staining assay (Fig. 8c, d and Supplementary Fig. 9c–f). Accordingly, both FK228 and JNJ treatment increased the proportion of senescence-associated (SA) β-gal-positive cells (Fig. 8e).

The results prompted us to access the therapeutic efficiency of HDACi in vivo. To achieve that, CNE1-TetOn-Vector and CNE1-TetOn-LMP1 cells were injected subcutaneously into athymic nude mice and expression of LMP1 was induced by Dox administration. Then the mice were divided into two groups, which were treated with either FK228 or vehicles (Fig. 8f). Treatment with FK228 was performed when tumors were clearly detectable, and thereafter, tumor volume was measured every 2 days until some mice displayed tumor-associated morbidity and were euthanized. Remarkably, tumor growth in CNE1-TetOn-LMP1 side was substantially suppressed by FK228 treatment, whereas tumor growth in CNE1-TetOn-Vector side showed little repression (Fig. 8g, h). To confirm whether the tumor growth inhibition is caused by FK288 induced differentiation in CNE1-TetOn-LMP1 cells, we stained the tumor tissues with antibodies against differentiation markers. We found that although LMP1 expression blocked the differentiation of NPC-derived cells, FK288 treatment almost completely reversed the LMP1 effects and induced the cellular differentiation of CNE1-TetOn-LMP1 cells (Fig. 8i). Comparable results were also observed in the JNJ treatment group (Supplementary Fig. 9g–i). Together, these data underlined the therapeutic potentials of FK228 for LMP1-positive poorly differentiated NPC in the clinic.

## Discussion

**Fig. 9:**
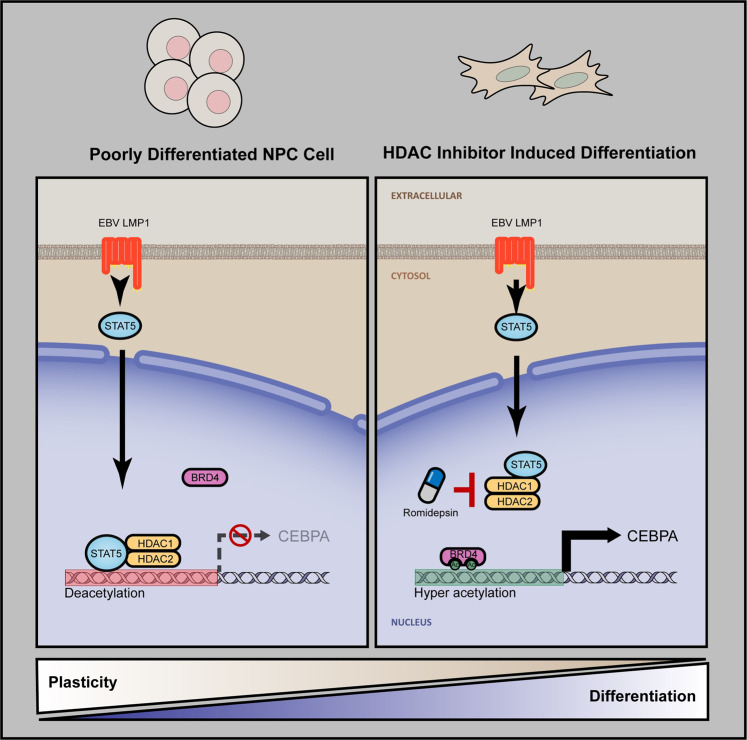
Model depicting the mechanism by which the HDAC inhibition reverses LMP1 suppressed differentiation in NPC cells

EBV associated undifferentiated nasopharyngeal carcinomas present serious therapeutic problems, including local relapse and distant organ metastases.^44^ Accumulated evidence supports that expression of LMP1 is strongly oncogenic in human epithelial cells.^37^ Nevertheless, whether expression of LMP1 drives the NPC cellular plasticity has hitherto remained uncertain. As summarized in Fig. 9, our findings demonstrate that EBV LMP1 inhibits the cellular differentiation program and endows cancer cell with aberrant cellular plasticity by epigenetic dysregulation. Mechanistically, expression of LMP1 epigenetically suppresses the transcription of CEBPA, an essential regulator of differentiation during development. Notably, CEBPA restoration by HDACi treatment reverses the LMP1-induced dedifferentiation and aberrant cellular plasticity in NPC cells.

Whether the HDAC inhibitor approach is likely to be useful in those LMP1 negative cases is worth further study. In LMP1 negative cells, HDACs might be recruited to *CEPBA* promoter and silence its transcription in a LMP1/STAT5A-independent mechanisms. Thus, HDACi would be useful in those LMP1 negative cases. In fact, a similar mechanism has been found in pioneer studies which found that the somatic mutations of NF-κB signaling’s negative regulators activate NF-κB pathway in LMP1 negative NPC tumors.^27,45^ Furthermore, HDACi has been shown to functionally inhibit the oncogenic NF-κB pathway,^46,47^ thus HDACi might also be useful in these distinct LMP1 negative NF-κB positive subclass cases.^27^

Recent clinical study established gemcitabine plus cisplatin as the standard first-line treatment option for recurrent or metastatic NPC.^48^ However, there is still a lack of target therapy for NPC. More effective and safer therapy remains to be explored. Here, we describe the US FDA-approved HDACi Romidepsin (FK228) and Chinese CFDA-approved HDACi Epidaza (Chidamide) restored protein level of CEBPA in NPC-derived cells expressing LMP1. The in vivo mice xenografts studies also support that HDACi would be effectively induce differentiation and reduce the cellular plasticity and stemness of NPC cells. Thus, our study opens up opportunities for epigenetic-based differentiation-inducing therapy in solid tumors. In fact, addition of the HDACi Chidamide, to the conventional chemotherapy regimen for two patients with poorly differentiated advanced NPC, led to sustained disease complete responses, supporting a potential role for HDACi in inhibiting cancer stemness and preventing disease progression (unpublished data); however, due to the limited sample size, no vigorous statistical conclusion can be drawn. Yet, this preliminary clinical study presents a justification for further, more controlled studies in larger patient groups. A large cohort is now under preparation to clarify the efficacy of addition of the HDACi Chidamide to conventional chemotherapy for progression-free survival in poorly differentiated advanced NPC.

The biological effects and potential therapeutic efficacy of HDAC inhibition has extensively been studied in hematopoietic differentiation and malignancies.^49–52^ HDACi, such as Romidepsin, Vorinostat, and Chidamide have been approved for clinical treatment of cutaneous T-cell lymphoma, peripheral T-cell lymphoma, and multiple myeloma.^53–56^ The current pilot study of Chidamide in NPC patient has extended the therapeutic potential of HDACi to solid tumors, advocating the further clinical studies of Romidepsin and the other HDACi approved by the US FDA or Conformit Europe (CE) in these patients.

CEBPA has been well documented as a master regulator in hematopoietic differentiation and as a silenced tumor suppressor in hematopoietic malignancies.^57^ Specifically, in acute myeloid leukemia (AML), CEBPA is transcriptionally repressed by AMLl-ETO and PML-RARa via recruiting co-repressors and histone deacetylases (HDACs).^58^ In agreement, ectopic expression of CEBPA restored defective neutrophil development.^59^ In a variety of solid tumors, CEBPA expression is repressed and its downregulation correlates with poor prognostic outcome; however, the precise mechanism remains unknown.^60^ A recent inspiring study demonstrates that CEBPA, as being one of the most TGF-β-mediated repressed transcription factors, is a master epithelial “gatekeeper” by preventing epithelial-to-mesenchymal switch in human mammary epithelial cells.^61^ Likewise, our study reveals that the ectopic expression of CEBPA restores NPC differentiation, echoing the “gatekeeper” role of CEBPA in epithelial tissues. Additionally, we find that the STAT5A/HDAC-mediated histone deacetylation at its promoter is a novel mechanism for CEBPA downregulation in NPC-derived cells. Another interesting study has reported that NF-κB transcriptionally actives CEBPA expression.^62^ However, in our study, although NF-κB is activated by LMP1, the STAT5/HDAC-mediated transcription repression is predominantly at the *CEBPA* promoter level, suggesting that histone acetylation is necessary for CEBPA activation in NPC.

STAT5, including the highly homologous proteins STAT5A and STAT5B, has also been described to play important roles in hematopoietic and epithelial differentiation and malignancies.^63–65^ However, the precise molecular mechanism lying downstream of STAT5 remains to be clarified. A previous study found that STAT5A recruits HDAC1 and activates the transcription of inhibitor of differentiation-1 (ID-1) via deacetylating transcription factor CEBPB in murine pro-B cells.^66^ Consistently, we found that STAT5A recruits HDAC1 and HDAC2 in NPC-derived cells. Whether STAT5B also plays a similar role in the signal axis warrants further investigation. Specifically, we uncovered that the target of HDACs is acetylated histone H3 at *CEBPA* promoter, and that histone H3 deacetylation by HDACs leads the transcriptional inhibition of CEBPA in NPC-derived cells. The discrete modification substrates and transcriptional regulation of STAT5-recruited HDACs appears to be tissue-specific or chromatin locus-specific. The molecular mechanism responsible for the substrate selectivity of HDACs warrants further investigation.

Collectively, our work provides a novel mechanistic epigenetic-based insight into EBV-induced cellular plasticity of NPC and further provides a prospective basis for promising clinical application of HDACi in virus-associated solid tumor differentiation therapy.

## Experimental section

### Cell lines

The immortalized normal human nasopharyngeal epithelial cell line NP69, EBV positive NPC cell line C666-1 and HK1-EBV were gifted by professor Musheng Zeng (Sun Yat-sen University). HK1, CNE1, HNE2, HONE1, and CNE2 were kindly gifted by Dr Chaonan Qian (Sun Yat-sen University). The STR and HPV integration of these cells were profiled and compared it with published data.^67^ The results are shown in Supplementary Table 1. CNE1, HNE2, HONE1, HK1, and CNE2 were cultured in RPMI 1640 (Invitrogen, C11875500BT) supplemented with 10% fetal bovine serum (Gibco, 12483020). C666-1 was cultured in RPMI 1640 supplemented with 20% FBS. NP69 cells was cultured in keratinocyte/serum-free medium (Invitrogen, MEPI500CA). All cells were cultured at 37 °C in a humidified chamber with 5% CO_2_.

### Lentivirus production and establishment of stable cell lines

pLVX-TRE3G-LMP1-IRES-EGFP, pLVX-TRE3G-LMP1, pLVX-TRE3G-LMP1-CEBPA-FLAG, pLVX-TRE3G-Vector, pLVX-TET3G, or pLKO-shLMP1 together with helper plasmids (pMD2G and psPAX2) were transfected into 293T cells using Lipofectamine 2000 transfection reagent, the transfection medium was replaced with fresh DMEM after 16 hours, and incubated for an additional 48 hours. The viral supernatants were collected through a 0.45 μm filter. CNE1, HNE2, and CNE2 cells were co-transduced with expression plasmids (pLVX-TRE3G-LMP1-IRES-EGFP, pLVX-TRE3G-LMP1, pLVX-TRE3G-LMP1-CEBPA-FLAG, or pLVX-TRE3G-Vector) and pLVX-Tet-3G viral supernatants at a ratio of 1:1. At 24 hours post infection, medium was freshed, followed by co-selection with G418 (Selleck, s3028) and puromycin (Selleck, s7417) after 48 hours for 1 week.

### Chemicals and antibodies

HDAC inhibitors TSA (S1045), JNJ-26481585 (S1096), FK228 (S3020), MGCD0103 (S1122), Chidamide (S8567), MS-275 (S1053), Tubacin (S2239), RGF966 (S7229), TMP269 (S7324) and BRD4 inhibitor JQ-1(S7110), JNK inhibitor SP600125(S1460), NF-κB inhibitor BAY 11-7082(S2913), MEK inhibitor PD98059 (S1177), and PI3K inhibitor Wortmannin (S2758) were purchased from Selleck Chemicals. Doxycicline was obtained from sigma (D9891). The following primary antibodies were used: LMP1 (DAKO, M0897), CEBPA (Cell Signaling Technology, 8178S), STAT5A (Santa Cruz Biotechnology, sc-1081), E-cadherin (Cell Signaling Technology, 3195 S), Phospho-STAT5 (Tyr694) (Cell Signaling Technology, 9314), CK8 (Proteintech, 17514-1-AP), CK14 (Protein Tech, 60320-1-Ig), Vimentin(Abcam, ab8978), Histone 3 (Abcam, ab130740), Acetylation Histone3 (Merck Millipore, 2604774), Histone4 (Abcam, ab8523), Acetylation Histone4 (Cell Signaling Technology, 9672), COX2 (Protein Tech, 12375-1-AP), p65 (4764S, Cell Signaling Technology), p50 (Beyotime, AF1246), CD44 (Proteintech, 15675-1-AP), p63 (Beyotime, AF1993), Nanog (Abcam, ab109250), SOX2 (Beyotime, AF8034), OCT4 (Beyotime, AF2506), AKT (Protein Tech, 60203-2-lg), phosphorylation-AKT (Cell Signaling Technology, 4060 S), ERK (Cell Signaling Technology, 4695), phospho-ERK(Cell Signaling Technology, 9107), Ki67 (BD, 610969), Goat anti-mouse (Thermo, 31430), and Goat anti-rabbit (Protein Tech, sa00001-2).

### Plasmid constructs and transfection

pCMMP-LMP1-IRES-EGFP was a gift from Bill Sugden (Addgene, plasmid # 36955). LMP1-IRES-EGFP were subcloned into Dox-inducible Lenti-viral expression Vector pLVX-TRE3G from the Lenti-X Tet-On 3G Inducible Expression System (Clontech, 631187). IRES-EGFP fragment in pLVX-TRE3G-LMP1-IRES-EGFP was deleted by using restriction enzyme EcoRI and ligation to get pLVX-TRE3G-LMP1. CEBPA-FLAG was generated by PCR and subcloned to pLVX-TRE3G-LMP1-IRES-EGFP by using the In-Fusion HD system (Clontech, 639636). pLKO-LMP1 was constructed by inserting the sequence targeting5′-ggaatttgcacggacaggc-3′ in LMP1 transcript as previously report.^68^ CEBPA transcription reporter was generated by PCR amplification from genomes of CNE1 cells and subcloned into the pGL3-basic luciferase Vector (Promega, E1751). Mutant construct was developed using the Site-Directed Mutagenesis (Clontech, 630701). The plasmid of STAT5A (WT) was provided by Zijie Long (Sun Yat-sen University, Guangzhou, China). Expression plasmids were transfected into cells using Lipofectamine 2000 (11668019, Invitrogen) according to the manufacturer’s instructions.

### RNA interference

The siRNAs were purchased from GenePharma. siRNAs transfection were performed following the user manual. The target sequences of siRNAs were listed in Supplementary Table 2.

### RNA-seq and quantitative RT-qPCR

For both cell lines and patients’ tumor tissues and non-tumor nasopharyngeal tissues, total RNA was isolated using TRIZOL reagent (Invitrogen, 1029602). The basic clinical information of patients is shown in Supplementary Table 3. RNA-seq was performed by Novogene company using Illumina HiSeq2000 (150 bp, paired-end). RNA-seq data was analyzed using RNACocktail.^69^ For quantitative RT-qPCR, RNA was reverse transcripted using EasyScript One-Step gDNA Removal and cDNA Synthesis SuperMix reverse transcription kit (Transgen, AT311) following the manufacturer’s instructions. Quantitative RT-qPCR assays were performed using Platinum SYBR Green qPCR SuperMix (Life, 4473908) as recommended by the manufacturer. The primers are listed in Supplementary Table 2.

### Dual-luciferase reporter assays

Dual-Luciferase Reporter Assay system (Promega, E1910) was used to determine the transcription activity according to the manufacturer’s instructions. The activity of Firefly and Renilla luciferase were measured sequentially. The promoter activity was calculated as the ratio of firefly luciferase activity to Renilla luciferase activity.

### Chromatin immunoprecipitation

Chromatin immunoprecipitation (ChIP) was performed using ChIP-IT Express Chromatin Immunoprecipitation Kits (Active Motif, 53008). CNE1 cells were crosslinked with 1% formaldehyde for 15 min at RT and then quenched with 10 ml 200 mM Glycine. The cross-linked chromatin was sheared using Covaris S220 (Covaris) following the user manual. Pre-cleared chromatin was then used for immunoprecipitation with 2 μl specific antibodies against acetylate Histone3, acetylate Histone4, STAT5, HDAC1, HDAC2, or control IgG and Protein G magnetic beads (25 μl) at 4 °C overnight. The immune complexes were washed, eluted, and reverse cross-linked according to the manufacturer’s protocol. DNA was extracted by phenol and phenol/chloroform extractions. The human *CEBPA* promoter-specific primers are listed in Supplementary Table 2.

### Biotinylated DNA pull-down assay

DNA pull down assay was performed as previous described.^70^ Briefly, CNE1-TetOn-LMP1 and control cells were treated with Doxycycline for 48 hours. Collected 3 × 10^7^ cells were washed twice by cold PBS. The cells were resuspended in ice-cold PBSI (PBS buffer containing protease inhibitors: 0.5 mM PMSF, 25 mM β-glycerophosphate, and 10Mm NaF). Then the cytoplasm was disintegrated by 2 package of buffer A (10 mM HEPES, pH 7.9, 1.5 mM MgCl2, 10 mM KCl, 300 mM sucrose, and 0.5% NP-40) with protease inhibitors for 10 min on ice. Vortex briefly, and centrifuge at 2600×*g* for 30 s. Remove supernatant and resuspend the pellet in 2/3 package cell volume of buffer B (20 mM HEPES, pH 7.9, 1.5 mM MgCl2, 420 mM NaCl, 0.2 mM EDTA, and 2.5% glycerol) with protease inhibitors. Sonicate the mixture for 5 min. Centrifuge at 10,400×*g* for 5 min. Then measure concentration of protein by Bradford Assay. Add 2 drops of the streptavidin-agarose bead suspension to a mixture of 400 μg of nuclear extract proteins and 4 μg of double-strand biotinylated oligonucleotides in 500 μl of PBSI buffer. Place the mixture on a rocking platform at 4 °C and rock the mixture at a gentle speed overnight. The immunocomplexes were pelleted, washed for multiple cycles at 4 °C, then subjected to SDS–PAGE and Western blotting analysis. The biotinylated double-stranded oligonucleotides are listed in Supplementary Table 2.

### Cytoplasmic and nuclear protein extraction

The cell pellets were resuspended in Buffer I (1 mM DTT, 25 mM HEPES pH 7.9, 5 mM KCl, 0.5 mM MgCl2) for 5 min to extract cytoplasmic protein. Next, the samples were incubated with Buffer II (25 mM HEPES pH 7.9, 5 mM KCl, 0.5 Mm MgCl_2_, 1 mM DTT and 0.4% (v/v) NP-40, protease inhibitors) and rotated at 4 °C for 15 min. The lysates were centrifuged at 500×*g* for 5 min at 4 °C. The supernatants were transferred to new Eppendorf tubes. Next, the pellets were rinsed once with Buffer II and centrifuged again for 5 min at 4 °C at 10,000×*g*, to remove the residual nuclei. To collect the nuclear extracts, the pellets were resuspended with Buffer III (25 mM HEPES pH 7.9, 400 mM NaCl, 10% sucrose or dextrose, 0.05% NP-40 and 1 mM DTT, and protease inhibitors) and rotated for 1 hour at 4 °C. The lysates were then centrifuged for 10 min at 4 °C at 10,000×*g* to remove the insoluble residue.

### Immunoblotting

Cells were washed twice with cold PBS and then lysed in RIPA buffer (50 mM Tris HCl pH 7.4, 150 mM NaCl, 1% NP-40, 0.5% sodium deoxycholate, 0.1% SDS, and 1 mM phenylmethyl sulfonyl fluoride) with complete protease (HY-K0011, MedChem Express) and phosphatase inhibitor cocktail (HY-K0021, MedChem Express) for 30 min. Lysates were collected and centrifuged at 12,000×*g* for 15 min to remove the insoluble pellets. Protein was quantified by Bradford Assay. Sample proteins were separated by SDS–PAGE gel electrophoresis. Next, proteins were transferred from gel to a nitrocellulose membrane (10600001, Millipore). After that, membranes were blocked in 5% milk TBST for 60 min at room temperature and then incubated with indicated primary antibodies overnight at 4 °C. Next, membranes were washed three times by TBST and incubated with peroxidase-conjugated secondary antibodies. Membranes were detected using an ECL detection kit (Advansta, 141104-03). The band intensities were determined using ImageJ software and the relative intensities were calculated as the ratios of sample band intensity to control band intensity.

The following antibodies were used: LMP1 (1:500 dilution), CK8 (1:1000 dilution), CK14 (1:1000 dilution), GAPDH (1:5000 dilution), CEBPA (1:1000 dilution), E-cadherin (1:1000 dilution), Vimentin (1:1000 dilution), Acetylation Histone3 H3 (1:1000 dilution), Histone4 H4 (1:1000 dilution), Acetylation Histone4 H4 (1:1000 dilution), COX2 (1:1000 dilution), p65 (1:1000 dilution), AKT (1:1000 dilution), phospho-AKT (1:1000 dilution), ERK (1:1000 dilution), phospho-ERK (1:1000 dilution), phospho-STAT5 (1:1000 dilution), p50 (1:1000 dilution), CD44 (1:1000 dilution), p63 (1:1000 dilution), Nanog (1:1000 dilution), SOX2 (1:1000 dilution), and OCT4 (1:1000 dilution). Goat anti-mouse and Goat anti-rabbit (1:5000 dilution) was used as a secondary antibody.

### Immunofluorescence staining

Cells were fixed with 4% paraformaldehyde at room temperature for 20 min and permeabilized with 0.5% Triton X-100 in PBS for 10 min. Then cells were incubated with primary antibodies overnight at 4 °C. Next, the cells were exposed to secondary antibodies conjugated to Alexa-488 Goat anti-Rabbit IgG (Invitrogen, A11008) Alexa-488 Donkey anti-mouse IgG (Invitrogen, A21202) or Goat anti-Mouse IgG Alexa-546 (Invitrogen, A21123), Goat anti-Rabbit IgG Alexa-546(Invitrogen, A11071) for one hour. Nuclear staining was performed using DAPI solution (Sigma, D9542).

### Immunoprecipitation

Cells for immunoprecipitation were freshly collected in cold PBS containing PMSF and lysed with RIPA Buffer supplemented with protease inhibitor cocktail (MedChem Express, HY-K0021). Subsequently, 300 μg of protein extract were incubated with 1 µg antibody at 4 °C for 1 hour with gentle rotation. Following 40 µl protein G-agarose was added and protein extracts were incubated overnight at 4 °C. For immunoblotting analysis, beads were washed three times with cold lysis buffer and then boiled in SDS sample buffer for immunoblotting analysis.

### SA-β-Gal staining

SA-β-Gal staining was performed by using Senescence β-Galactosidase Staining Kit (Beyotime, C0602). Cultured cells were washed with PBS and SA-β-Gal activity was determined according to the manufacturer’s directions.

### Colony formation assay

In total, 500 cells were seeded into six-well plates in triplicate then cultured for 10–14 days. Cells were cultured in medium containing 100 ng/ml Doxycycline to induce the expression of LMP1 or CEBPA. Colonies were stained with crystal violet and counted.

### Immunohistochemical staining

Paraffin-embedded tissue specimens were sectioned, deparaffinized in xylene and rehydrated. Antigenic retrieval was processed with sodium citrate. The sections were then incubated in H_2_O_2_ (3%) for 10 min, blocked in 1% bovine serum albumin for 60 min and incubated with indicated antibodies at 4 °C overnight. After incubation with the secondary antibody for 60 min, specimens were incubated with H_2_O_2_-diaminobenzidine until the desired stain intensity was developed.

### Methylation-specific PCR and bisulfite sequencing PCR

To perform *CEBPA* promoter methylation analysis, the bisulfite-modified DNA was PCR amplified with locus-specific primers. Bisulfite conversion of GC-rich DNA was performed by using EZ DNA Methylation-Gold^TM^ Kit (D5005, ZYMO RESEARCH) according to the manufacturer’s instructions. Products of methylation-specific PCR were analyzed by agarose gel electrophoresis. The products of bisulfite sequencing PCR were subcloned to T-Vector and sequenced. methylation level were analyzed by BiQ Analyzer (8). Primer sequences of human CEBPA for the unmethylated reaction, methylated reaction, and bisulfite sequencing are listed in Supplementary Table 2.

### Bioinformatics analysis

For STRING analysis (http://string-db.org/), candidate genes were subjected for analyzing with default analyzing parameters. Binding proteins on *CEBPA* promoter was obtained from JASPAR (http://jaspar.genereg.net/) on vertebrates. GSEA analysis were performed using GSEA v2.0.13 software (http://www.broad.mit.edu/gsea) with 1000 data permutations.

### Animal studies and primary cell isolation

For tumor growth assays, 6 × 10^6^ cells in PBS containing 50% Matrigel (Thermo, A1413301) were subcutaneously injected at the dorsal of nude mice (4–6 weeks). After the cell injection, expression of LMP1 or CEBPA were induced by oral administration of doxycycline delivered by water containing 1 mg/ml doxycycline and 1 g/ml sucrose. When the tumors reached 150 mm^3^ in volume, the mice were randomly separated into four groups and treated with HDACi (JNJ-26481585, FK228) via intraperitoneal injection. HDACi were prepared in a vehicle of 20% Hydroxypropyl-β-Cyclodextrin in PBS and were administered at a dose of 100 μg/kg JNJ or 120 μg/kg FK228. The body weight of the animals and the two perpendicular diameters (a and b) were recorded every 3 days. Tumor volume (V) was calculated according to the following formula: V = (a×b×b)/2. After 6 weeks, tumor mass was resected and dissociated for following experiments.

### Study approval

All animal studies and human subject research were approved by the ethical committee of Sun Yat-Sen University Cancer Center (IRB approval number GZR2013-110).

### Statistical analysis

All in vitro experiments were repeated at least three times and in vivo experiments twice. Statistical analysis of colonies and cell numbers, cell cycle, senescence, percent expression of markers associated with differentiation were carried out with directly measured data. Threshold cycle (Ct) of target mRNA from RT-qPCR was normalized with GAPDH mRNA Ct, then ddCt was calculated and the result presented as 2^-ddCt^.^71^ Statistical analysis was performed with Student’s *t*-test between two independent groups, one-way analysis of variance (ANOVA) with Bonferroni’s post-test among multiple groups. Data were analyzed using SPSS software and generated plots with GraphPad Prism 8. A *P* value of <0.05 was considered statistically significant. *P* values were indicated by asterisks as followed: *P < 0.05, **P < 0.01, ***P < 0.001, NS: no significance.

## Supplementary information

Supplementary Figures and Tables

## Data Availability

The raw sequence data reported in this paper have been deposited in the Genome Sequence Archive (Genomics, Proteomics & Bioinformatics 2017) in BIG Data Center (Nucleic Acids Res 2019), Beijing Institute of Genomics (BIG), Chinese Academy of Sciences, under accession numbers CRA002212 that are publicly accessible at https://bigd.big.ac.cn/gsa.
