# Supplementary material for: Targeting cancer cell plasticity by HDAC inhibition to reverse EBV-induced dedifferentiation in nasopharyngeal carcinoma
Source: Signal Transduct Target Ther. 2021 Sep 4;6:333. doi: 10.1038/s41392-021-00702-4 (PMC8418605; doi:10.1038/s41392-021-00702-4)
Supplement: Supplementary file 1 — Supplementary Figures and Tables [file 41392_2021_702_MOESM1_ESM.docx]

Supplementary Materials for

Targeting Cancer Cell Plasticity by HDAC Inhibition to Reverse EBV-induced Dedifferentiation in Nasopharyngeal Carcinoma

Jiajun Xie, Zifeng Wang, Wenjun Fan, Youping Liu, Fang Liu, Xiangbo Wan, Meiling Liu, Xuan Wang, Deshun Zeng, Yan Wang, Bin He, Min Yan, Zijian Zhang, Mengjuan Zhang, Zhijie Hou, Chunli Wang, Zhijie Kang, Wenfeng Fang, Li Zhang, Eric W-F Lam, Xiang Guo, Jinsong Yan, Yixin Zeng, Mingyuan Chen, and Quentin Liu

Correspondence to: Quentin Liu, liuq9@mail.sysu.edu.cn; Mingyuan Chen, chenmy@sysucc.org.cn; Yixin Zeng, zengyx@sysucc.org.cn; Jinsong Yan, yanjsdmu@126.com

**This PDF file includes:**

Figures. S1 to S9

Tables. S1 to S3


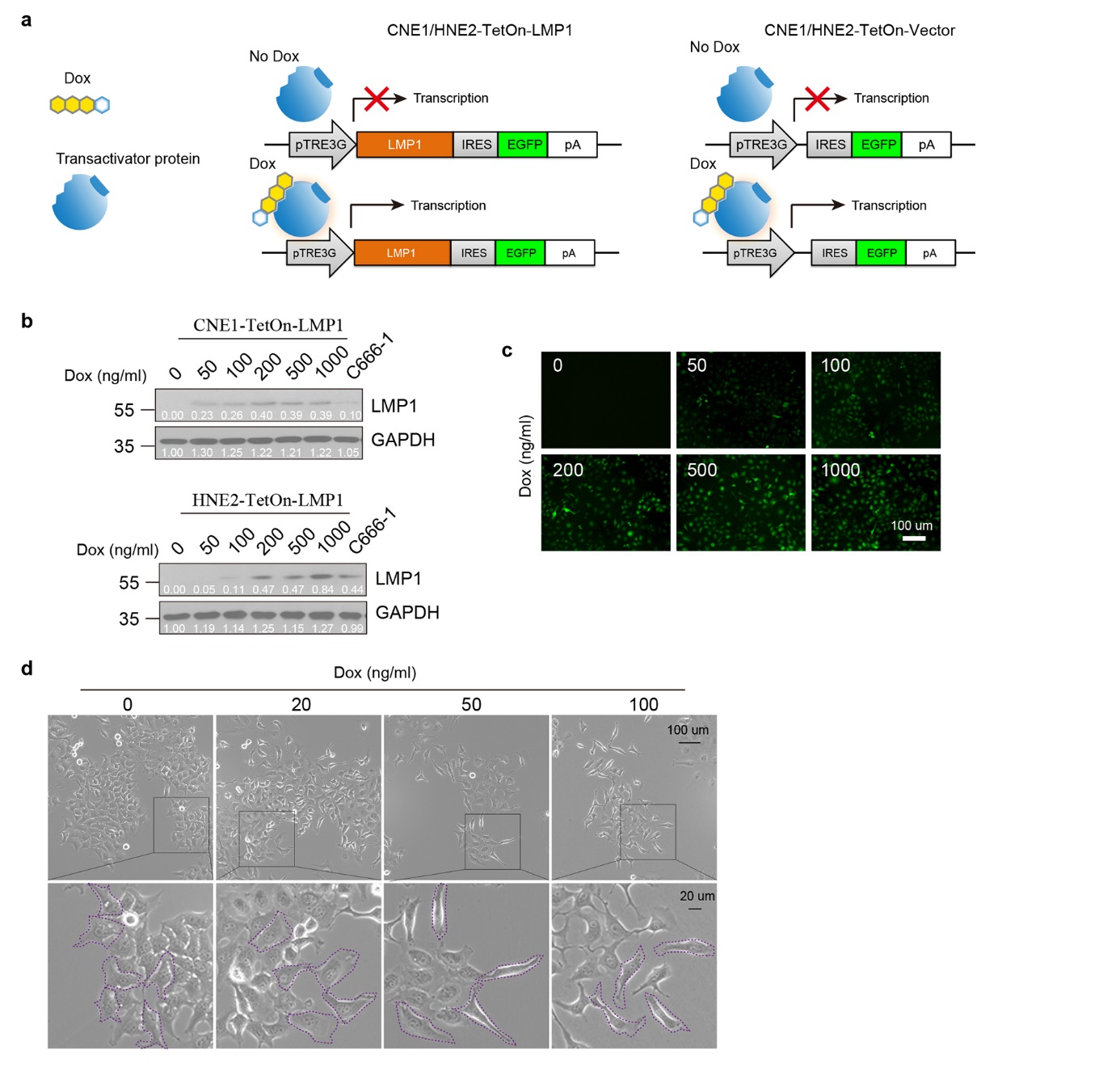


Figure. S1.

(a) Schematic of the inducible LMP1 expression systems for NPC-derived cells. (b) CNE1-TenOn-LMP1 and HNE2-TenOn-LMP1 cells were treated with a series of Dox concentration for 48 hours, and then cell lysates were subjected to Western blot analysis. (c) Fluorescence of EGFP in CNE1-TenOn-LMP1 cells treated with a series of Dox concentration for 48 hours. (d) Phase contrast images of CNE1-TetOn-LMP1 cells treated with a series of Dox concentration for 48 hours.


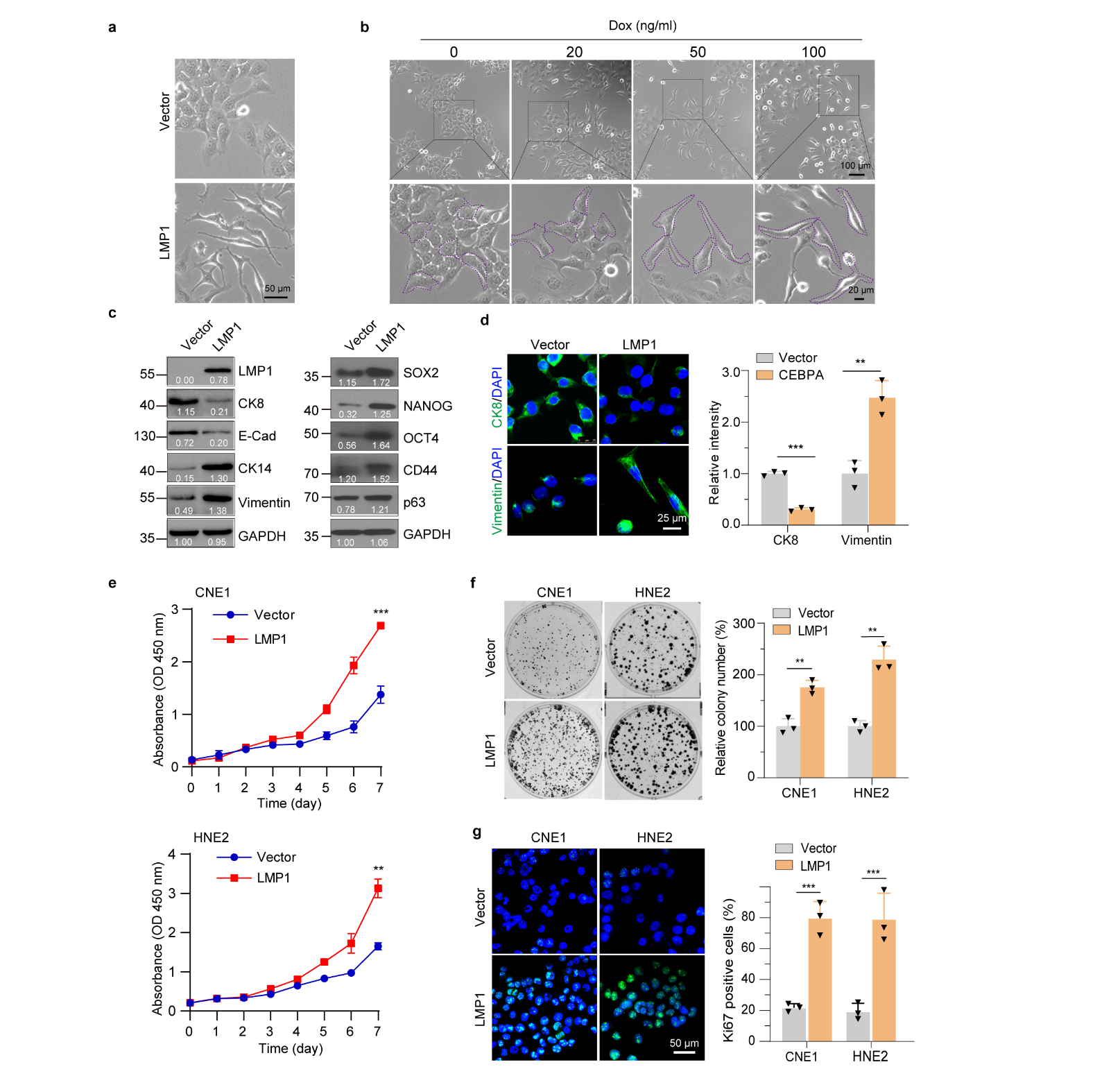


Figure. S2.

(a) Phase contrast images of HNE2-TetOn-Vector and HNE2-TenOn-LMP1 cells treated with 100 ng/ml Dox for 48 hours. (b) Phase contrast images of HNE2-TetOn-LMP1 cells treated with a series concentration of Dox for 48 hours. (c and d) Western blot and immunofluorescence staining with differentiation markers and stem-like markers in HNE2-TetOn-Vector and HNE2-TetOn-LMP1 cells treated with 100 ng/ml Dox for 48 hours. (e) CNE1/HNE2-TetOn-Vector and CNE1/HNE2-TetOn-LMP1 cells were treated with 100 ng/ml Dox, and then the cells proliferation abilities were measured using CCK8 assay. (f and g) CNE1/HNE2-TetOn-LMP1 cells and respective control cells were treated with 100 ng/ml Dox for 48 hours then subjected to colony formation and immunofluorescence staining for Ki67. Statistics (d-g), significance: ***P* < 0.01, ****P* < 0.001; two-tailed Student’s *t*-tests.


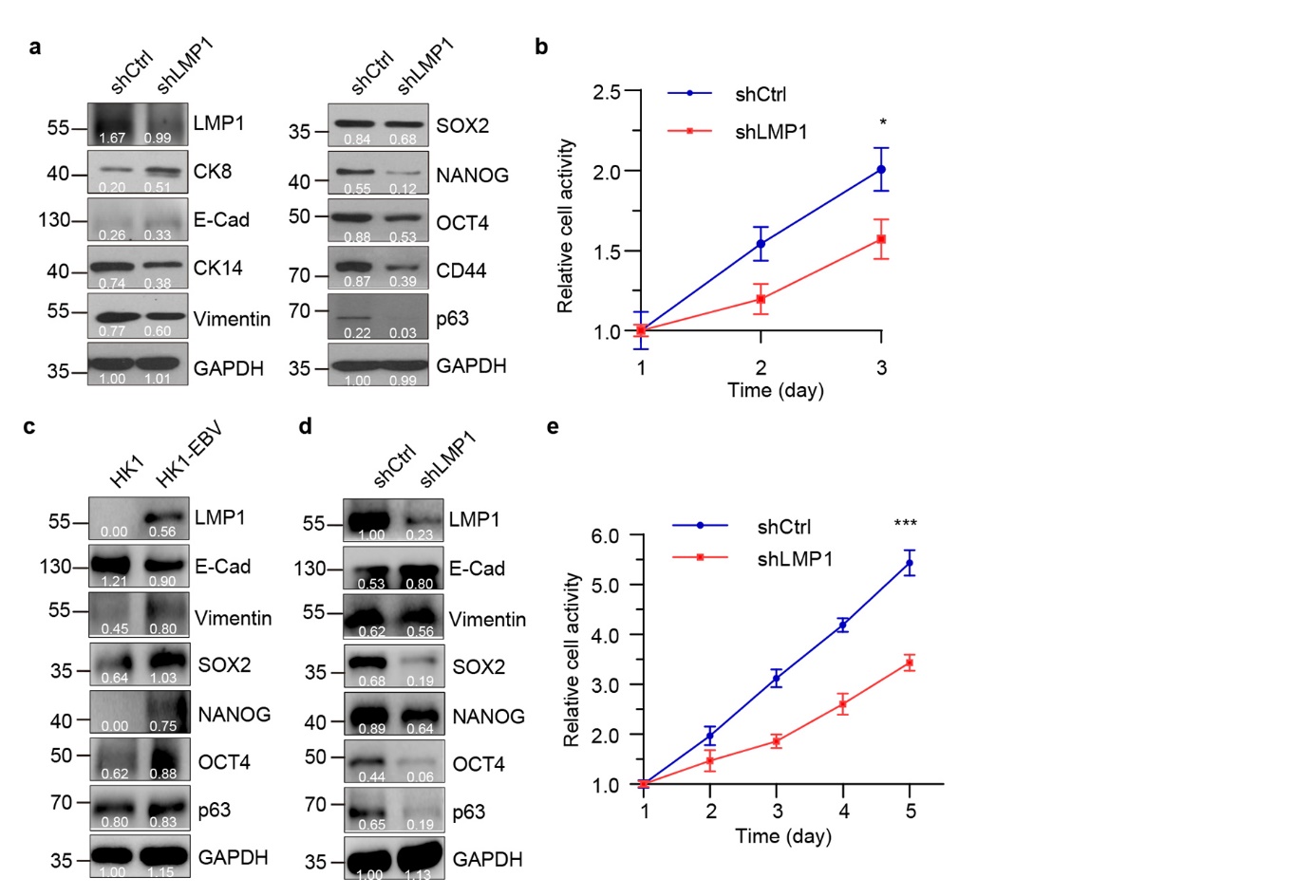


Figure. S3.

(a) Western blot with differentiation markers and stem-like markers in C666-1 cells with or without knockdown of LMP1. (b) C666-1 cells with or without knockdown of LMP1 were assayed for their cell proliferation ability using CCK8 assay. (c) Western blot with differentiation markers and stem-like markers in HK1 cells with or without EBV infection. (d) Western blot analysis with differentiation and stem-like markers in HK1-EBV cells with or without knockdown of LMP1. (e) The cell proliferation abilities were measured using CCK8 assay in HK1-EBV cells with or without knockdown of LMP1. Statistics (b and e), significance: **P <* 0.05, ****P <* 0.001; two-tailed Student’s *t-*test.


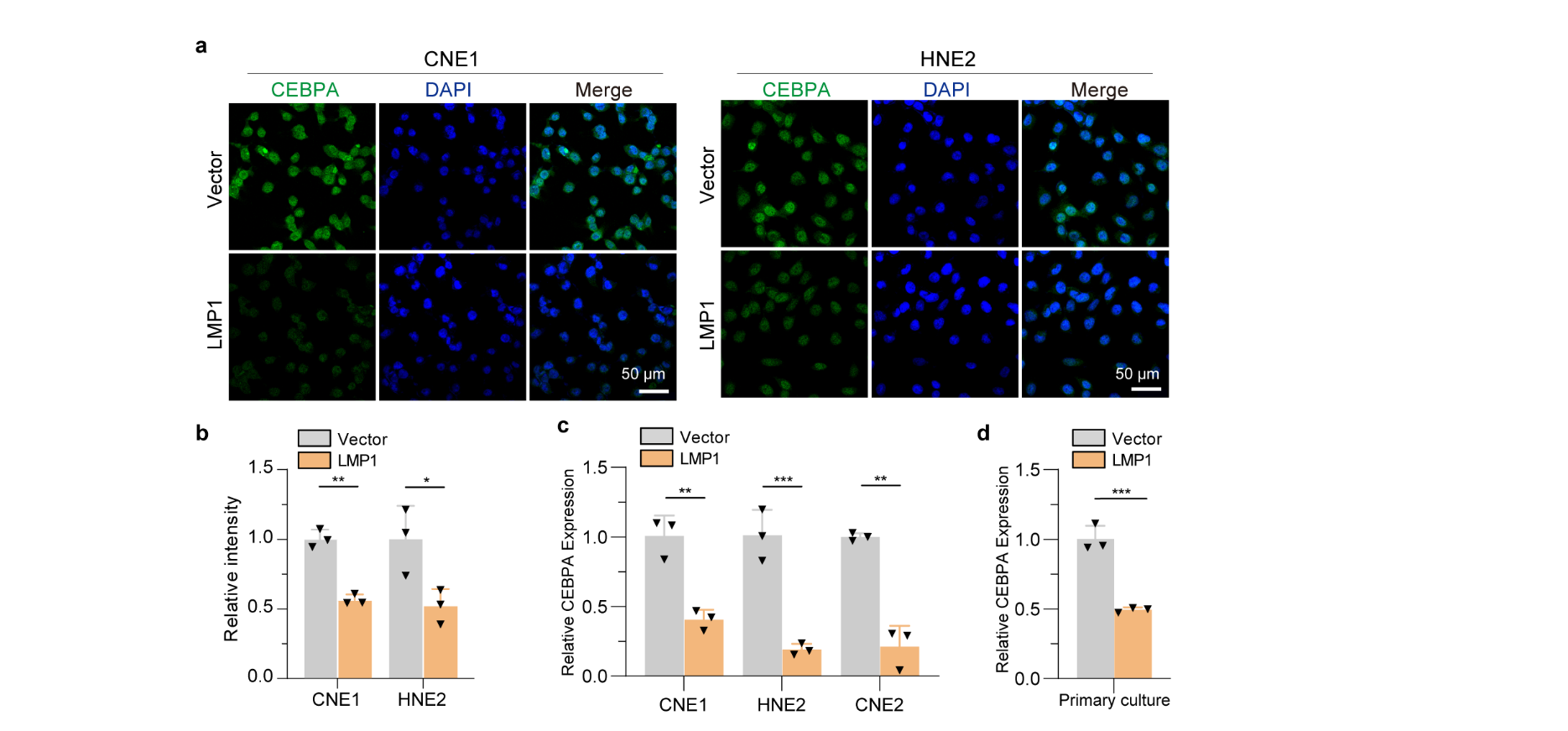


Figure. S4.

(a) Immunostaining of CEBPA in CNE1/HNE2-TetOn-LMP1 and CNE1/HNE2-TetOn-Vector cells after 100 ng/ml Dox treatment for 48 hours. (b) Statistics of immunostaining from (a). (c) RT-qPCR analysis of relative CEBPA mRNA levels in CNE1/HNE2/CNE2-TetOn-LMP1 and CNE1/HNE2/CNE2-TetOn-Vector cells after 100 ng/ml Dox induction of LMP1 for 48 hours. (d) RT-qPCR analysis of relative CEBPA mRNA levels in tumor cells with or without LMP1 induction. Statistics (b-d), significance: **P* < 0.05, ***P* < 0.01, ****P* < 0.001; two-tailed Student’s *t*-tests.


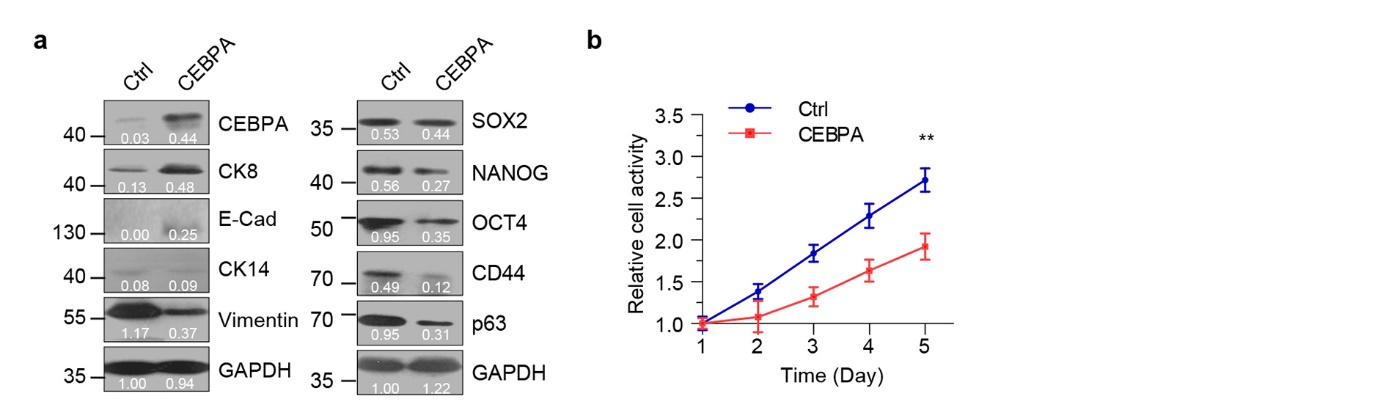


Figure. S5.

(a) Western blot analysis with differentiation markers and stem-like markers in C666-1 cells with or without CEBPA overexpression. (b) Proliferation ability of C666-1 cells with or without CEBPA overexpression was determined by CCK8 assay. Statistics, significance: ***P <* 0.01; two-tailed Student’s *t-*tests.


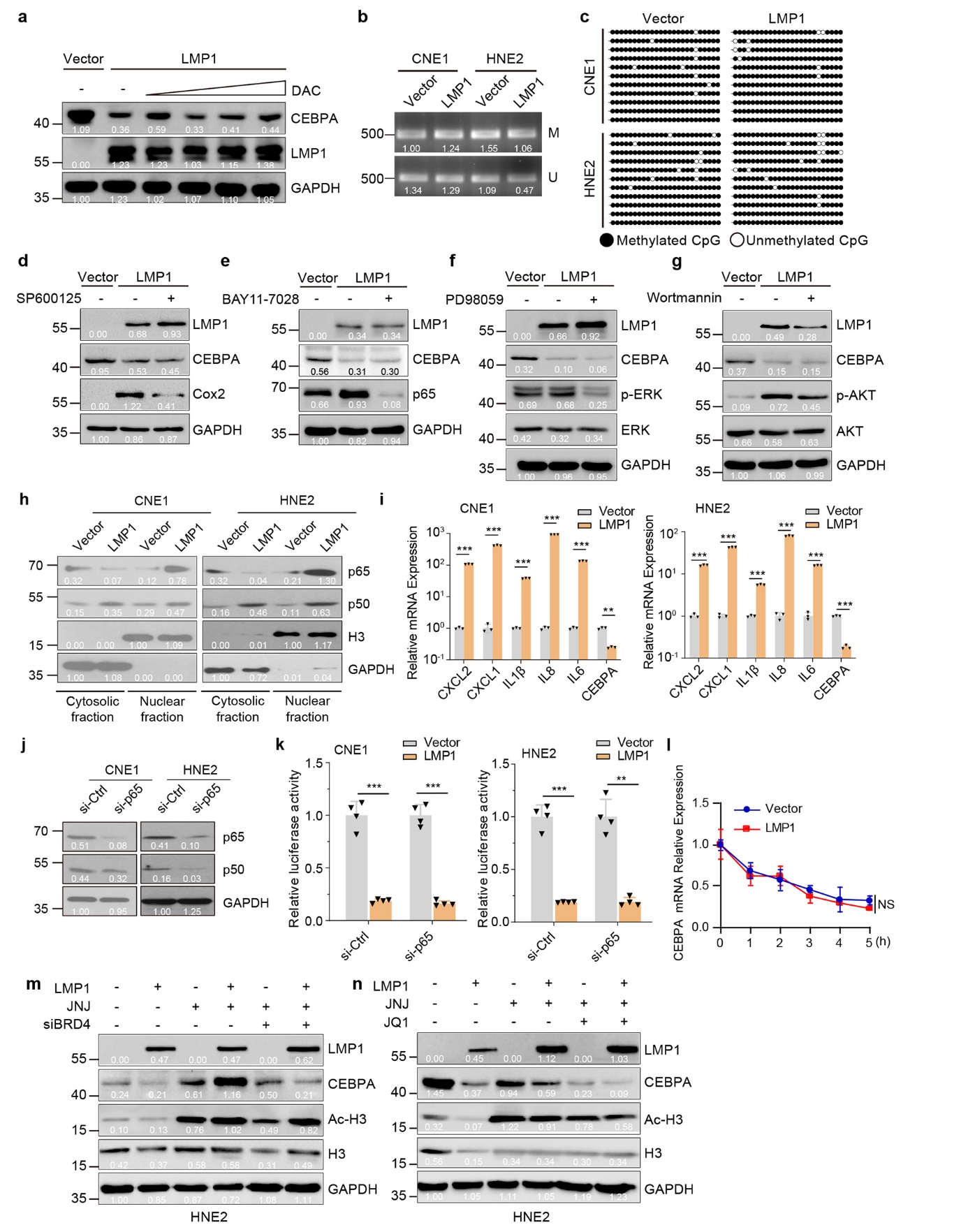


Figure. S6.

(a) CNE1-TetOn-LMP1 and CNE1-TetOn-Vector cells were treated with 100 ng/ml Dox for 48 hours, and then exposed to DNA methylation inhibitor DAC. Protein levels of CEBPA were determined by immunoblotting. (b and c) Methylation levels of CEBPA promoter were detected by methylation-specific PCR or bisulfite sequencing PCR in the indicated cells. (d-g) CNE1-TetOn-LMP1 and CNE1-TetOn-Vector cells were treated with 100 ng/ml Dox for 24 hours, and then exposed to 20 µM SP600125, 10 µM BAY11-7028, 50 µM PD98059 and 100 nM Wortmannin for further 24 hours, respectively. Then the indicated protein levels were determined by immunoblotting. (h) Expression and subcelluar distribution of p65 and p50 in CNE1/HNE2-TetOn-LMP1 and CNE1/HNE2-TetOn-Vector cells treated with 100 ng/ml Dox for 48 hours. (i) mRNA expression of NF-κB canonical targets CXCL2, CXCL1, IL1β, IL8 and IL6 was determined in CNE1/HNE2-TetOn-LMP1 and CNE1/HNE2-TetOn-Vector cells treated with 100 ng/ml Dox for 48 hours. (j) p65 was knockdown by siRNA in CNE1/HNE2-TetOn-LMP1 cells. Protein levels of p65 and p50 were determined by immunoblotting. (k) CEBPA promoter luciferase activity (-1424~0) in p65 knockdown and control cells with or without LMP1 expression was detected by dual-luciferase reporter assays. (l) The mRNA stability of CEBPA was determined in CNE1-TetOn-LMP1 and CNE1-TetOn-Vector cells after 100 ng/ml Dox induction. (m and n) Protein levels of CEBPA were determined by immunoblotting. Knockdown of BRD4 by siRNA or BRD4 inhibitor treatment in HNE2-TetOn-Vector and HNE2-TetOn-LMP1 cells with 100 ng/ml Dox induction for 48 hours, then cells were exposed to 200 nM JNJ. Statistics (i and k-l), significance: ***P <* 0.01, ****P <* 0.001; NS: no significance; two-tailed Student’s *t-*tests.


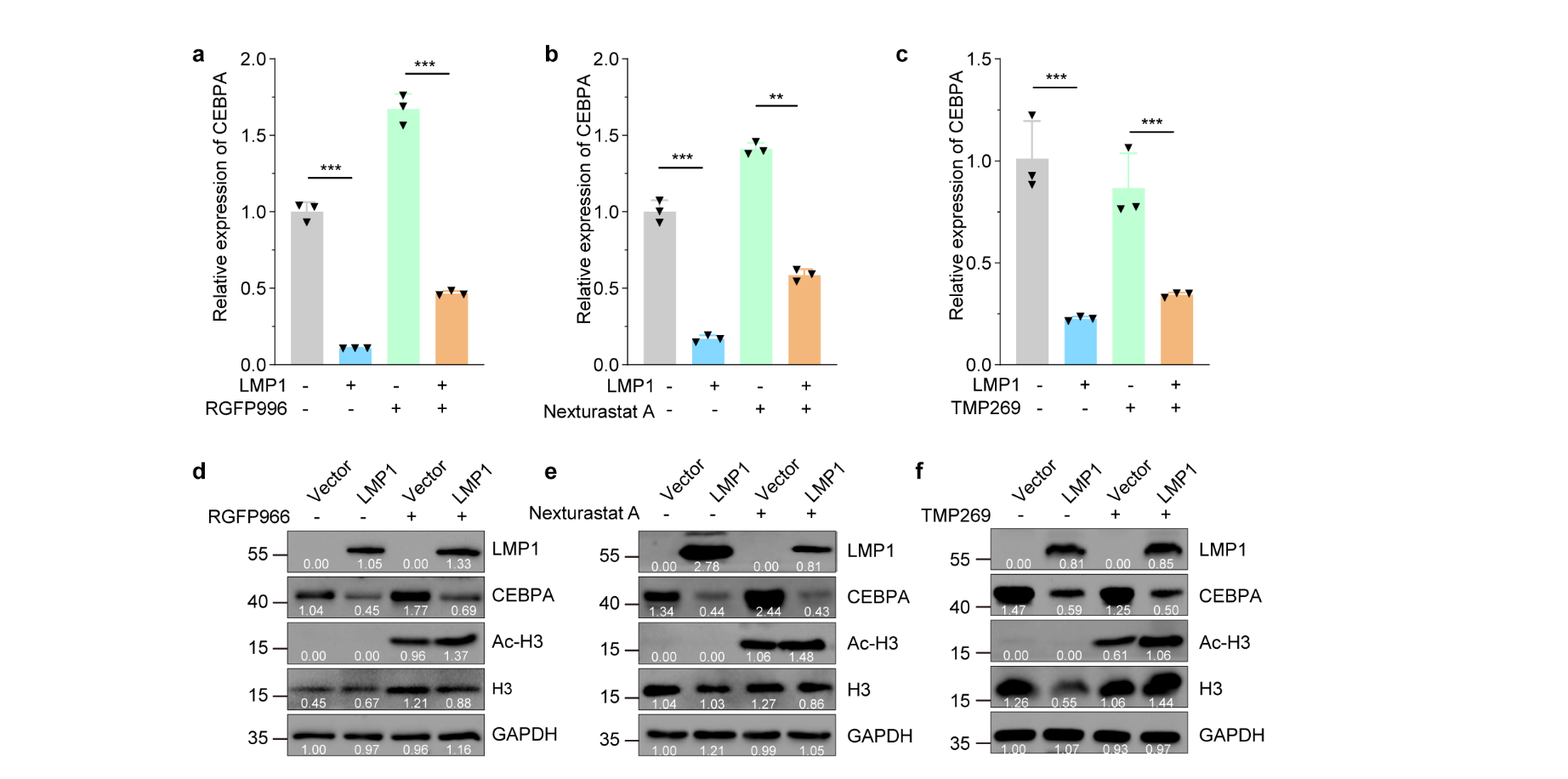


Figure. S7.

(a-c) CNE1-TetOn-LMP1 and CNE1-TetOn-Vector cells were treated with 100 ng/ml Dox for 24 hours, and then exposed to 5µM RGFP996, 10 µM Nexturastat A and 10 µM TMP269 for further 24 hours, respectively. Then mRNA levels of CEBPA were determined by RT-qPCR. (d-f) CNE1-TetOn-LMP1 and CNE1-TetOn-Vector cells were treated with 100 ng/ml Dox for 24 hours, and then exposed to 5 µM RGFP996, 10 µM Nexturastat A and 10 µM TMP269 for further 24 hours, respectively. The cellular protein levels of CEBPA were determined by immunoblotting. Statistics (a-c), significance: ***P <* 0.01, ****P <* 0.001; two-tailed Student’s *t-*tests.


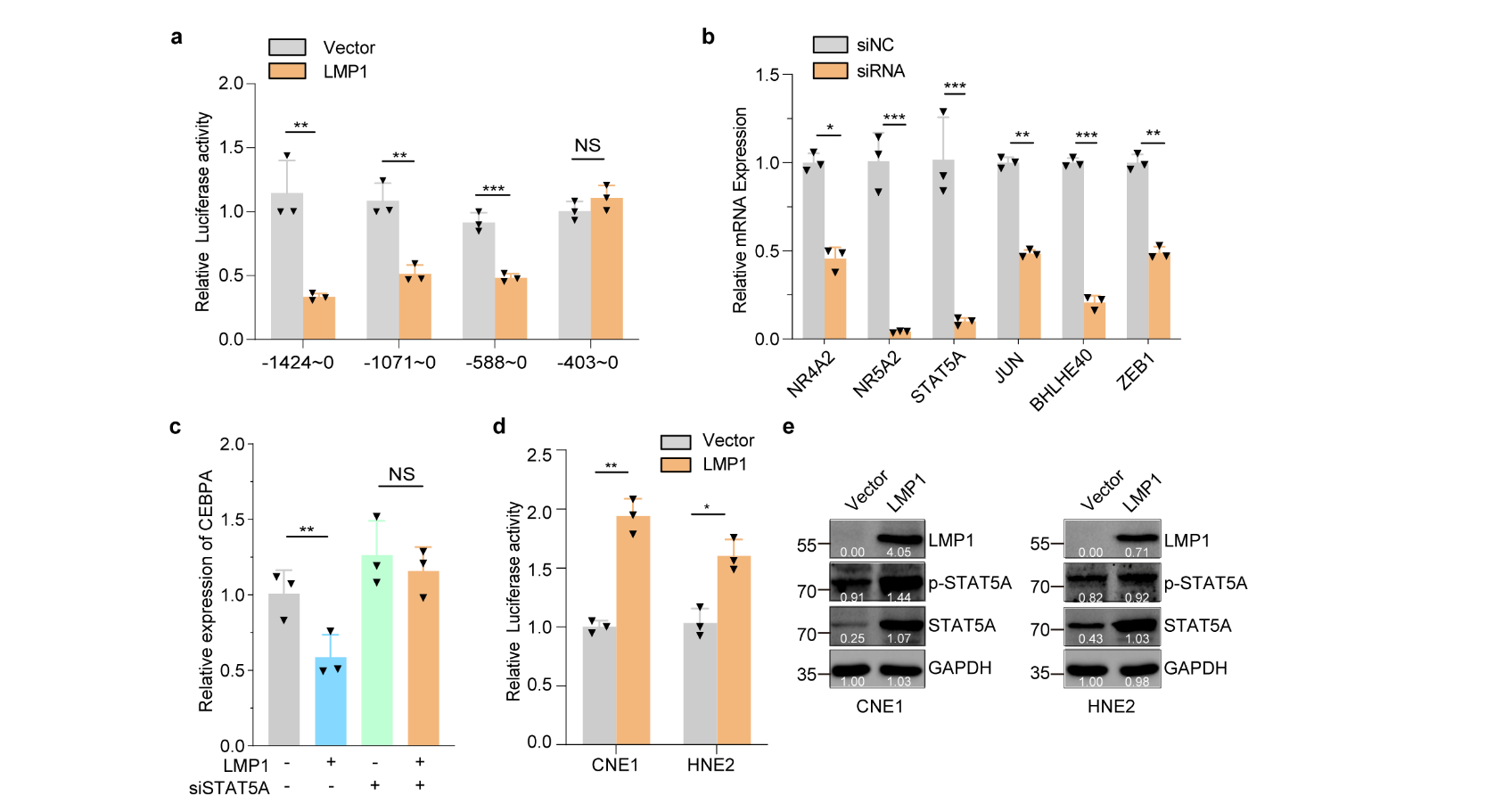


Figure. S8.

(a) Truncated CEBPA promoter luciferase activity in HNE2-TetOn-LMP1 and HNE2-TetOn-Vector cells were detected by dual-luciferase reporter assays. (b) Knockdown efficiency of siRNAs was determined by RT-qPCR. (c) CNE1-TetOn-LMP1 and CNE1-TetOn-Vector cells were transfected with STAT5A siRNAs or negative control for 48 hours. mRNA level of CEBPA were determined by RT-qPCR. (d) STAT5A promoter luciferase activity in CNE1/HNE2-TetOn-LMP1 and CNE1/HNE2-TetOn-Vector cells were detected by dual-luciferase reporter assays. (e) CNE1/HNE2-TetOn-LMP1 and CNE1/HNE2-TetOn-Vector cells were collected and subject to immunoblot with STAT5A antibody. Statistics (a-d), significance: **P <* 0.05, ***P <* 0.01, ****P <* 0.001, NS: no significance; two-tailed Student’s *t-*test.


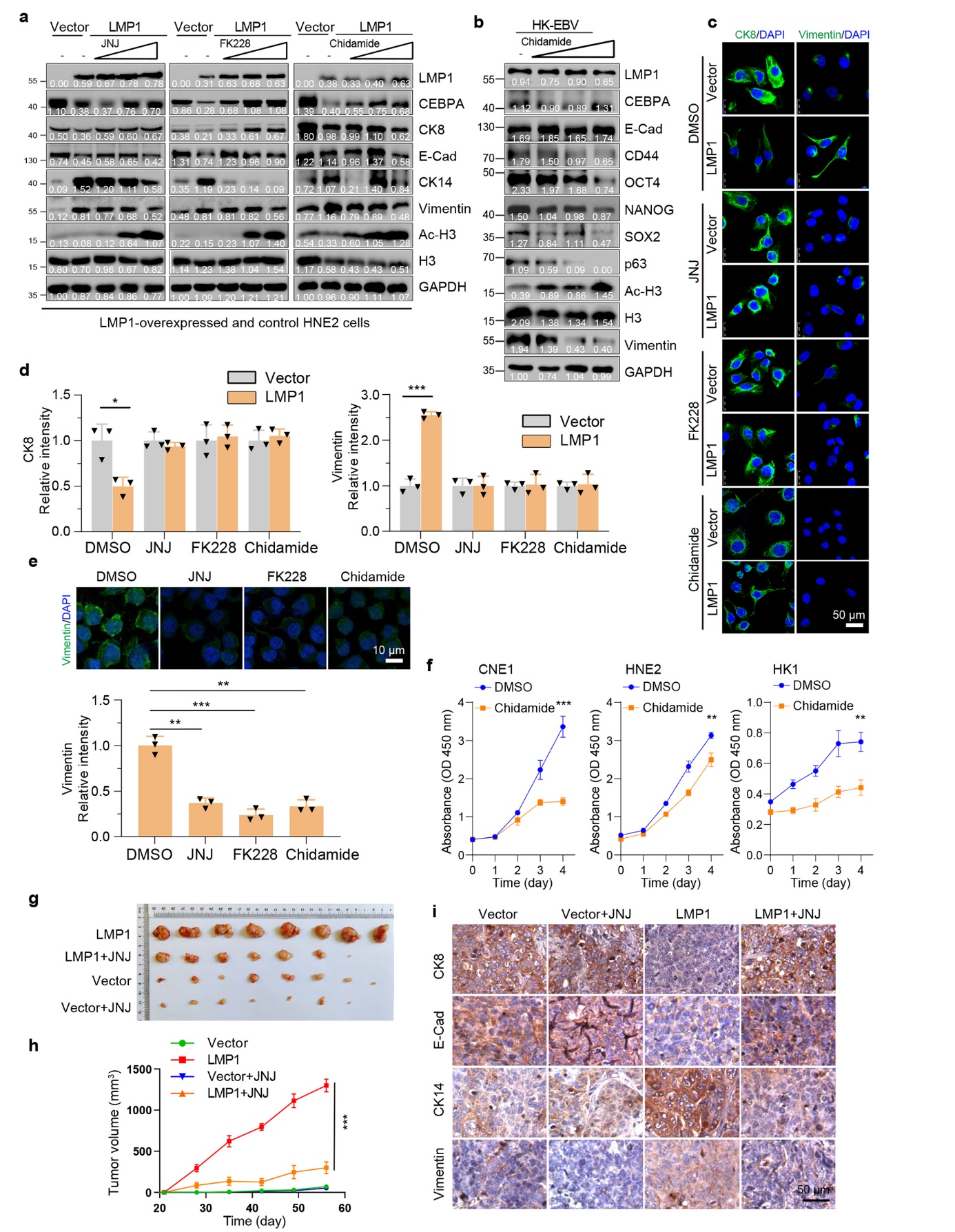


Figure. S9.

(a) HNE2-TetOn-LMP1 and HNE2-TetOn-Vector cells were treated with JNJ (0, 2, 20, and 200 nM), FK228 (0, 0.5, 5, and 50 nM), or Chidamide (0, 0.5, 1, and 3 μM). Expression of CEBPA, differentiation markers and stem-like markers were examined by immunoblotting. (b) HK1-EBV cells were treated with Chidamide (0, 0.5, 1, and 3 μM). Expression of CEBPA, differentiation markers and stem-like markers were examined by immunoblotting. (c-e) HNE2-TetOn-LMP1 and HNE2-TetOn-Vector cells, or HK1-EBV cells were treated with200nM JNJ, 10 nM FK228, or 1 μM Chidamide for 48 hours and immunofluorescence staining was performed with indicated antibodies. (f) LMP1-overexpressed CNE1, HNE2, or HK1 cells were treated with Chidamide then the cell proliferation ability was measured using CCK8 assay. (g and h) The images of dissected tumors at the endpoints of the experiments were shown and growth curves were plotted by measuring the relative tumor volumes at indicated days. (i) Immunohistochemistry was performed in tumors from (g) for differentiation markers. Statistics, significance: **P <* 0.05, ***P <* 0.01, ****P <* 0.001; two-tailed Student’s *t-*test (d, f, and h); one-way ANOVA with Bonferroni correction (e).

Table S1.

STR profiles and HPV status of cell lines


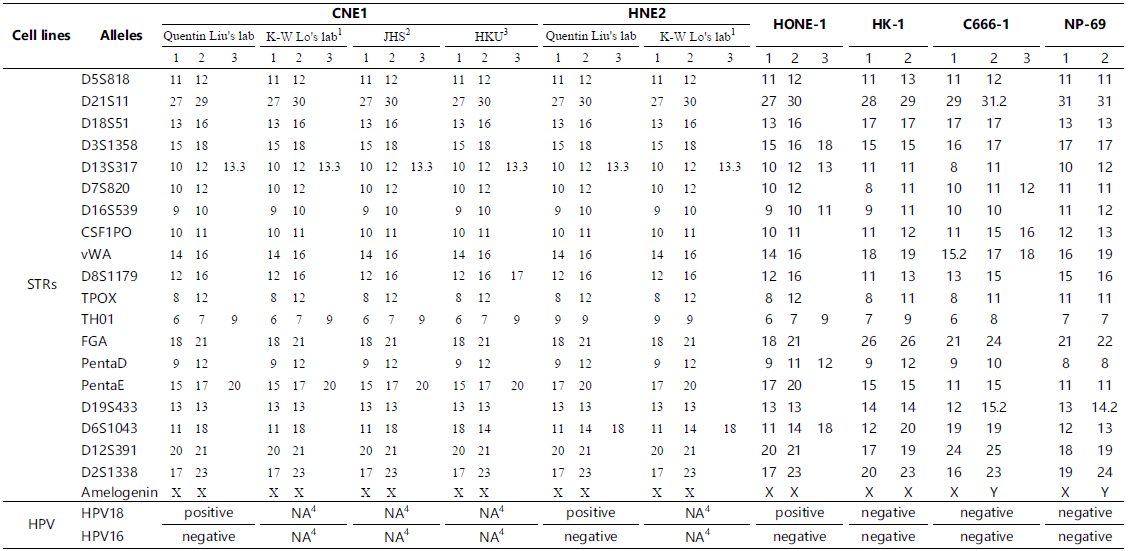


^1^K-W Lo's lab, the lab of professor Kwok-Wai Lo in The Chinese University of Hong Kong; ^2^JHS, Johns Hopkins Singapore; ^3^HKU, The University of Hong Kong; ^4^NA, not available.

Table S2.

Sequences of siRNAs, primers, and oligonucleotides

| Sequences of siRNAs |  |
| --- | --- |
| Name | **siRNA Targets** |
| siBRD4  siHDAC1  siHDAC2 | CCUGAUUACUAUAAGAUCATT  GCUCCUCUGACAAACGAAUTT  CCAUGAAGCCUCAUAGAAUTT |
| siNR4A2 | GGCGAACCCUGACUAUCAATT |
| siNR5A2 | GCACGGACUUACACCUAUUTT |
| siSTAT5A #1  siSTAT5A #2  siJUN  siBHLHE40  siZEB1 | GCGCUUUAGUGACUCAGAATT  GCUGGCUAAAGCUGUUGAUTT  GGCACAGCUUAAACAGAAATT  CAAACCUAAUUGAUCAGCATT  GCUACUGGAGAUGGCAAUUTT |

| Sequences of RT-qPCR primers |  |
| --- | --- |
| Name | **Sequences** |
| CEBPA-F | TGGACAAGAACAGCAACGAGT |
| CEBPA-R | CAGGCGGTCATTGTCACTGG |
| AQP3-F | GGGGAGATGCTCCACATCC |
| AQP3-R  PPARG-F  PPARG-R  BMP4-F  BMP4-R  THRA-F  THRA-R  HES1-F  HES1-R  ZNF488-F  ZNF488-R  TP73-F  TP73-R  FGFR3-F  FGFR3-R  CA2-F  CA2-R  NR4A2-F  NR4A2-R  NR5A2-F  NR5A2-R  STAT5A-F  STAT5A-R  JUN-F  JUN-R  BHLHE40-F  BHLHE40-R  ZEB1-F  ZEB1-R | AAAGGCCAGGTTGATGGTGAG  ACCAAAGTGCAATCAAAGTGGA  ATGAGGGAGTTGGAAGGCTCT  TAGCAAGAGTGCCGTCATTCC  GCGCTCAGGATACTCAAGACC  AGGTCACCAGATGGAAAGCG  AGTGATAACCAGTTGCCTTGTC  TCAACACGACACCGGATAAAC  GCCGCGAGCTATCTTTCTTCA  GAAAACAGATGGCGACTTAGCG  CTGCCGGTCCTTCATCCTC  CCACCACTTTGAGGTCACTTT  CTTCAAGAGCGGGGAGTACG  TGCGTCGTGGAGAACAAGTTT  GCACGGTAACGTAGGGTGTG  GGGTACGGCAAACACAACG  GGCTGTATGAGTGTCGATGTC  GTTCAGGCGCAGTATGGGTC  CTCCCGAAGAGTGGTAACTGT  CTTTGTCCCGTGTGTGGAGAT  GTCGGCCCTTACAGCTTCTA  GCAGAGTCCGTGACAGAGG  CCACAGGTAGGGACAGAGTCT  TCCAAGTGCCGAAAAAGGAAG  CGAGTTCTGAGCTTTCAAGGT  GACGGGGAATAAAGCGGAGC  CCGGTCACGTCTCTTTTTCTC  GCTGTTGCTGATGTGGCTTT  GGTCATCCTCTGGTACACCT |
| IL6-F | ACTCACCTCTTCAGAACGAATTG |
| IL6-R | CCATCTTTGGAAGGTTCAGGTTG |
| IL8-F | ACTGAGAGTGATTGAGAGTGGAC |
| IL8-R | AACCCTCTGCACCCAGTTTTC |
| IL1β-F | AGCTACGAATCTCCGACCAC |
| IL1β-R | CGTTATCCCATGTGTCGAAGAA |
| CXCL1-F | GAAAGCTTGCCTCAATCCTG |
| CXCL1-R | CTTCCTCCTCCCTTCTGGTC |
| CXCL2-F | GGGCAGAAAGCTTGTCTCAA |
| CXCL2-R | GCTTCCTCCTTCCTTCTGGT |
| GAPDH-F | TCAACGACCACTTTGTCAAGCTCA |
| GAPDH-R | GCTGGTGGTCCAGGGGTCTTACT |

| Sequences of ChIP RT-PCR and ChIP RT-qPCR primers | |
| --- | --- |
| Name | **Sequences** |
| CEBPA promoter-specific primers-F  CEBPA promoter-specific primers-R  STAT5A binding site-F | CCCCCGCTACCGACCACGTG  TCCTAGCGCGCGGCCGGCATG  TAGGAGACGCAGAGCCACC |
| STAT5A binding site-R | CTTTCAAAGCCAGAACCAG |
| Negative Ctrl site-F | CCATCCACAGCCTCAGGAAGAA |
| Negative Ctrl site-R | TGGGAGCAAAGGAGAAATCACC |

| Sequences of biotinylated double-stranded oligonucleotides of CEBPA promoter | |
| --- | --- |
| Name | **Sequences** |
| CEBPA promoter- F | biotin-CTCTGCGCTGCAGCCTCCCCGGGACGCGGGTCCGGG |
| CEBPA promoter-R | biotin-CCCGGACCCGCGTCCCGGGGAGGCTGCAGCGCAGAG |

| Sequences of methylation-specific PCR and bisulfite sequencing PCR primer | |
| --- | --- |
| Name | **Sequences** |
| CEBPA promoter-unmethylated reaction-F  CEBPA promoter-unmethylated reaction-R  CEBPA promoter-methylated reaction-F  CEBPA promoter-methylated reaction-R  CEBPA promoter-bisulfite sequencing-F  CEBPA promoter-bisulfite sequencing-R | AGTGTGTTTTTAGTTGGGATGTAG  AAACTCTACCCAATAATTCACCAA  AGGTAAGCGCGTTTTTAGTCGG  TCCCCGAAATCCTAAAAACCGAA  TTGTTAGGTTTAAGGTTATTG  AAACCCTAAAACCCCTTA |

Table S3.

Clinical information of patient samples

| **Variable** | **Inflammation** | **NPC** |
| --- | --- | --- |
|  | *n* (%) | *n* (%) |
| **Gender** |  |  |
| Female | 3 (30.0) | 3(30.0) |
| Male | 7 (70.0) | 7 (70.0) |
| **Age** (Median + SD (range)) | 41.5 ± 15.3 (15-65) | 55.2 ± 9.1 (40-72) |
| **Karnofsky performance status** |  |  |
| ≥ 90 | 10 (100) | 10 (100.0) |
| ＜90 | 0 (0) | 0 (0) |
| **T classification** |  |  |
| T1 | NA**^*^** | 0 (0) |
| T2 | NA | 1 (10.0) |
| T3 | NA | 4 (40.0) |
| T4 | NA | 2 (20.0) |
| Unknown | NA | 3 (30.0) |
| **N classification** |  |  |
| N1 | NA | 5 (50.0) |
| N2 | NA | 1 (10.1) |
| N3 | NA | 1 (10.0) |
| Unknown | NA | 3 (30.0) |
| **M classification** |  |  |
| M0 | NA | 7 (100) |
| M1 | NA | 0 (0) |
| Unknown | NA | 3 (30.0) |
| **Overall stage** |  |  |
| I | NA | 0 (0) |
| II | NA | 1 (10.0) |
| III | NA | 4 (40.0) |
| IV | NA | 2 (20.0) |
| Unknown | NA | 3 (30.0) |
| **Histology** |  |  |
| WHO I | NA | 0 |
| WHO II | NA | 0 |
| WHO III | NA | 10 (100) |
| **Plasma EBV DNA copies** |  |  |
| > 0 | 2 (20.0) | 6 (60.0) |
| 0 | 8 (80.0) | 3 (30.0) |
| Unknown | 0 (0) | 1 (10.0) |
| **LMP1 status^#^** |  |  |
| Positive | 0 (0) | 10 (100) |
| Negative | 10 (100) | 0 (0) |

^*^NA, not available. ^#^LMP1 positive is defined as the cycle threshold (Ct) values of LMP1 in RT-qPCR assays are greater than 32; LMP1 negative is defined as the Ct values of LMP1 in RT-qPCR assays are less than 32.
